# Novel Technique for Simultaneous Ethylene Glycol and Its Metabolites Determination in Human Whole Blood and Urine Samples Using GC–QqQ–MS/MS

**DOI:** 10.3390/jox14030065

**Published:** 2024-08-27

**Authors:** Kaja Tusiewicz, Olga Wachełko, Marcin Zawadzki, Paweł Szpot

**Affiliations:** 1 Department of Forensic Medicine, Wroclaw Medical University, 4 J. Mikulicza-Radeckiego Street, 50345 Wroclaw, Poland; 2 Institute of Toxicology Research, 45 Kasztanowa Street, 55093 Borowa, Poland; 3 Faculty of Medicine, Department of Social Sciences and Infectious Diseases, Wroclaw University of Science and Technology, 27 Wybrzeże Wyspiańskiego, 50370 Wrocław, Poland

**Keywords:** MTBSTFA derivatization, PFPH derivatization, ethylene glycol intoxication, GC–QqQ–MS/MS, postmortem biological material

## Abstract

Toxicological analyses often necessitate the identification of compounds belonging to diverse functional groups. For GC–MS analyses, derivatization of compounds belonging to different functional groups can pose a challenge and requires the development of comprehensive methods of analysis. One example could be ethylene glycol, whose widespread use is related to possible unintentional or suicidal intoxications. This fact clearly indicates the need to develop sensitive methods for the determination of ethylene glycol and its metabolites in biological material, as only such complex analysis allows for proper toxicological expertise. A simultaneous GC–QqQ–MS/MS method for the determination of ethylene glycol together with its metabolites, glyoxal and glycolic acid, as well as the detection of glyoxylic acid and oxalic acid, was developed and fully validated. A novel approach for simultaneous derivatization of substances from different groups (alcohols, aldehydes, and carboxylic acids) was established. Sample preparation included the addition of three internal standards (BHB-*d_4_*, ethylene glycol-*d_4_* and methylglyoxal), precipitation with acetonitrile and subsequent derivatization with *N*-*tert*-butyldimethylsilyl-*N*-methyltrifluoroacetamide (MTBSTFA), as well as pentafluorophenylhydrazine (PFPH). Detection was carried out with the use of triple quadrupole mass spectrometer. The ionization method was electron impact, and quantitative analysis was carried out in multiple reaction monitoring mode. The lower limit of quantification was 1 μg/mL, 0.1 μg/mL, and 500 μg/mL for ethylene glycol, glyoxal, and glycolic acid, respectively. The presented method was applied in three authentic postmortem cases of ethylene glycol intoxication.

## 1. Introduction

Chemical derivatization is often an indispensable element of the pre-analytical phase in the determination of many chemical compounds using gas chromatography coupled with mass spectrometry (GC–MS). This necessity arises from a series of features provided by derivatization in the context of this analytical technique, such as increasing compound volatility and stability, improving recovery during extraction, enabling better chromatographic separation, and enhancing detector sensitivity and detection selectivity by increasing the analyte’s mass [1,2]. The choice of reagent used for derivatization is closely related to the functional group present in the analyte’s chemical structure [1,2]. In the case of more complex chemical compounds, it may happen that there are two different functional groups eligible for derivatization, or there may be a need for simultaneous determination of compounds with different functional groups. Such a scenario provides the opportunity to apply simultaneous or parallel derivatization reactions of several different functional groups, as exemplified by the determination of steroid hormones [3] or compounds from five different chemical groups [4]. These exemplary reactions on non-biological material can potentially be transferred to the preparation of biological samples for GC–MS analysis. One example of a situation in forensic toxicology where such simultaneous analysis of compounds from different structural groups could be necessary is the determination of ethylene glycol and its metabolites, which contain hydroxyl, carboxyl, and aldehyde groups.

The estimated production of ethylene glycol worldwide is believed to reach more than 23 billion kg per year. Its main use, due to its ability to lower the freezing point of a solution, is the production of antifreeze, which is added to brake, hydraulic, and radiator fluids, as well as fluids for washing cars used in the automotive industry [5]. Such widespread use of this compound is related to possible unintentional or occupational exposure resulting in intoxication. What is more, easy access to substances that can be bought even online, e.g., as azides [6] or cyanides [7,8] kits, as well as organic solvents like chloroform [9] or ethylene glycol [10], might serve for suicidal purposes.

**Figure 1:**
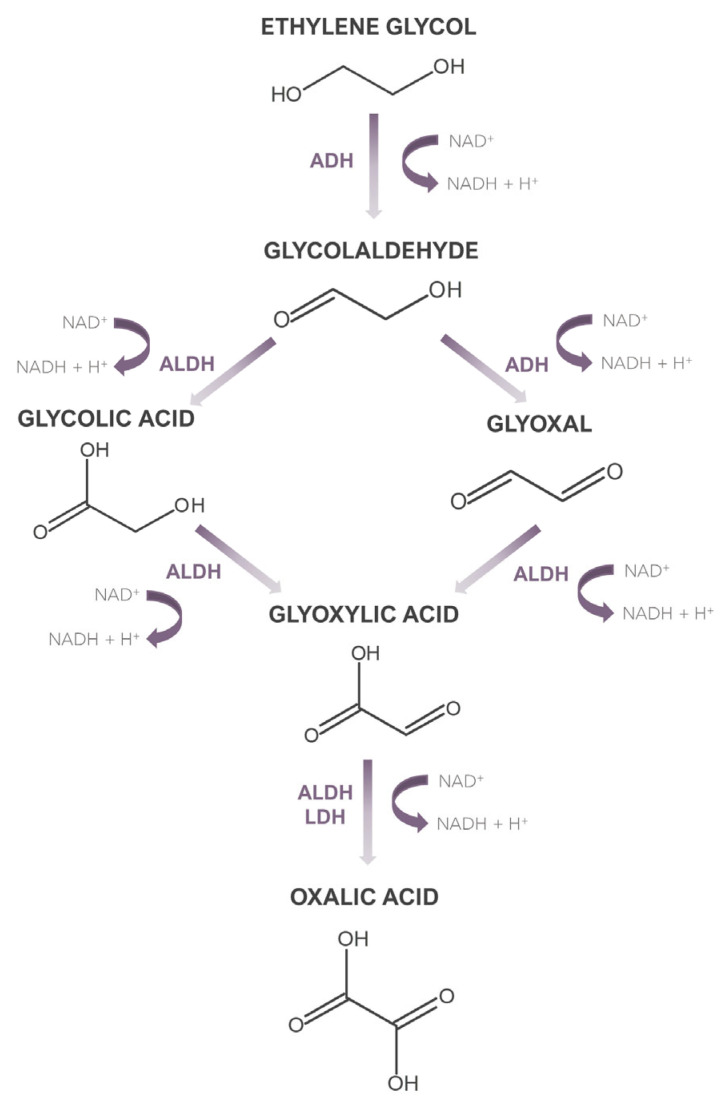
Metabolism of ethylene glycol.

In 2007, the American Poison Control Centre reported 5395 cases of ethylene glycol intoxication [11], which clearly indicates the need to develop accurate and sensitive methods for the determination of ethylene glycol in biological material. However, as the vast majority of methods applied to date have focused only on determining ethylene glycol, it is equally important to have the possibility of determining the metabolites of this compound. This is dictated by the fact that ethylene glycol, when absorbed into the body in its native form, does not exhibit high toxicity, unlike its metabolites. Moreover, it should be noted that in some cases, when dealing with the late phase of intoxication, ethylene glycol may be present in the sample at a very low concentration, which less sensitive methods will not determine. Then the detection of its metabolites can serve as a marker indicating potential ethylene glycol poisoning. What is more, determining the concentration of ethylene glycol metabolites can be helpful in determining the phase of intoxication with this xenobiotic, which can have important clinical significance. An ethylene glycol metabolism path is shown in Figure 1. The biological half-life of ethylene glycol is 3–8.6 h [12]. The metabolism of this xenobiotic occurs in the liver, and its first step is the conversion to glycolaldehyde, which is then converted into glycolic acid and glyoxal. Subsequent oxidation leads to the formation of glyoxylic acid, which is then converted to oxalic acid. The latter creates insoluble complexes with calcium, which are deposited in many organs, mainly the kidneys, leading to their damage [13]. Biochemical processes accompanying these transformations, along with accumulated glycolic acid, which is the metabolite present in the highest concentration during intoxication, are the cause of metabolic acidosis. The transformation of glycolic acid is considered the rate-limiting step in ethylene glycol metabolism, as the biological half-life of glycolic acid is 10 h [13].

Taking all the aforementioned issues into consideration, both ethylene glycol and its metabolites should be taken into account during the clinical diagnosis of intoxication and during toxicological expertise. This is due to the fact that they are an additional confirmation of ethylene glycol poisoning; they help to estimate the phase of intoxication; or, in the case where the entire parent compound has metabolized in time, they are the only confirmation of ethylene glycol exposure. However, to identify all metabolites of ethylene glycol, which hold the highest diagnostic value, simultaneous determination of compounds from the group of alcohols, aldehydes, and carboxylic acids is necessary.

From the literature review on the determination of ethylene glycol in biological material using GC–MS, it is apparent that only four authors have developed methods for the simultaneous determination of ethylene glycol and its metabolite, glycolic acid [14,15,16,17]. To the best knowledge of the authors, there is no reported method capable of determining ethylene glycol and a greater number of its metabolites in biological material. Individual papers have described the determination of separate ethylene glycol metabolites in biological material, but in a different context than ethylene glycol poisoning. Judea-Pusta et al. [18] mention in their paper that they determined glycolic acid using the GC method. Lapolla et al. [19] determined glyoxal in relation to diabetic patients by the GC–MS method. Ethylene glycol metabolites have also been independently determined in biological material in other contexts using techniques such as potentiometric titration and colorimetric methods [20,21] high-performance liquid chromatography with diode array detection [22], ultra-high-performance liquid chromatography with tandem mass spectrometry detection [23], and high-performance liquid chromatography with fluorescence detection [24,25,26,27,28].

Therefore, in this study, the authors aim to present a developed method for the simultaneous determination of compounds possessing different functional groups (alcohols, aldehydes, and acids), using ethylene glycol and its metabolites as an example.

### 1.1. Epidemiology of Ethylene Glycol Intoxications

**Figure 2:**
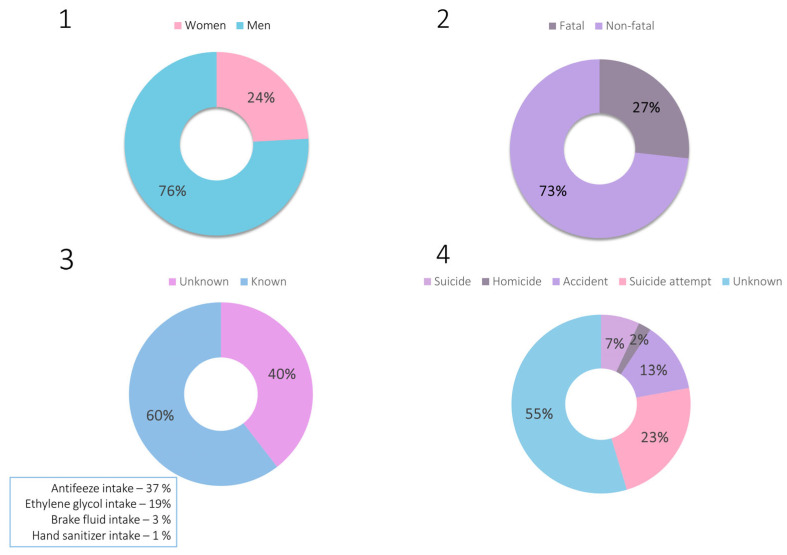
Charts representing epidemiological data on ethylene glycol intoxications: **1**—sex of the victims, **2**—outcome of the intoxication, **3**—route of intoxication, **4**—reason of intoxication.

Based on the available scientific literature on ethylene glycol intoxications, the epidemiology of intoxications with this xenobiotic was investigated using the Google Scholar database. The following phrase was searched: “ethylene glycol intoxication case”. Sixty-five papers were found (after discarding irrelevant or inaccessible ones), describing a total of 81 cases of poisoning dated from 1960 to 2024. The highlights of all cases are summarized in Supplementary Material S1, together with a detailed description of the epidemiological findings. The epidemiological data in the form of charts are also presented in Figure 2.

The gathered epidemiological data demonstrate that ethylene glycol poisonings constitute a significant aspect of both clinical and forensic toxicology. These are primarily accidental or suicidal poisonings, with the widespread availability of products containing this compound suggesting that such poisonings will continue to occur. In the analysis of the described poisoning cases, attention was also paid to the concentrations of ethylene glycol determined in biological material and to the indication of detecting its metabolites. Among the non-fatal cases, ethylene glycol concentrations were as follows: 145–7060 μg/mL (blood), 40–10,700 μg/mL (serum), and 80.5–13,600 μg/mL (urine). Postmortem blood ethylene glycol concentrations were as follows: 152–7800 μg/mL (blood), 425–7750 μg/mL (serum), whereas the concentration in urine was determined in only one case and equaled 10,200 μg/mL. It is also worth mentioning that only in two papers out of all published to date [18,29] has the concentration of glycolic acid been mentioned.

### 1.2. The Aim of the Work

The aim of the work was to develop a simultaneous GC–QqQ–MS/MS method for the determination of ethylene glycol together with its metabolites, glyoxal and glycolic acid, as well as qualitative detection of glyoxylic acid and oxalic acid by the use of a novel approach of simultaneous derivatization of compounds from the alcohols, aldehydes, and carboxylic acid groups. Furthermore, the developed method was then successfully applied to three authentic cases of ethylene glycol intoxication, enabling the determination of the abovementioned compounds in *postmortem* blood and urine samples.

## 2. Materials and Methods

### 2.1. Chemicals

The following reagents were used in the development of the method: water (LiChrosolv^®^ LC-MS), acetonitrile (LiChrosolv^®^ LC-MS), methanol (LiChrosolv^®^ LC-MS), *n*-hexane (LiChrosolv^®^ LC-MS, glyoxal solution (40 wt.% in water), methylglyoxal solution (40% in water), glycolic acid solution (70 wt.% in water), glyoxylic acid solution (50 wt.% in water), oxalic acid (purity > 99.0%), deuterated ethylene glycol-*d_4_* (98 atom%D), *N*-tert-butyldimethylsilyl-*N*-methyltrifluoroacetamide with 1% tert-butyldimethylchlorosilane (MTBSTFA), pentafluorophenylhydrazine (PFPH), hydrochloric acid 30% and anhydrous ethylene glycol were purchased from Sigma-Aldrich^®^ (Steinheim, Germany), deuterated sodium salt (10 mg) of 3-hydroxybutanoic acid (BHB-*d_4_*) was purchased from TRC (Toronto, ON, Canada).

### 2.2. Biological Material

Drug-free, blank whole blood samples used for the development and validation of the method were obtained from the Regional Blood Donation Center. Drug-free, blank urine sample collection was performed during autopsy. The approval of the bioethics committee required has been obtained (consent no. KB-184/2023). Blank samples (blood and postmortem urine) were screened prior to spiking to ensure that they were free from drugs. Authentic human whole blood and urine samples were sent to our laboratory for toxicological analyses for ethylene glycol intoxication.

### 2.3. Working Solutions, Calibration Curve, Quality Control Samples

The stock solutions and standard solutions were stored at −20 °C. Pentafluorophenylhydrazine was diluted in hexane to a concentration of 10 mg/mL. BHB-*d_4_* salt was dissolved in methanol to obtain a stock solution with a concentration of 10 mg/mL. Methylglyoxal solution was mixed with water to obtain a concentration of 10 mg/mL. Then, stock solutions of BHB-*d_4_,* ethylene glycol-d4, and methylglyoxal were mixed together with methanol to achieve a final concentration of 1 mg/mL BHB-*d_4_*, 1 mg/mL ethylene glycol, and 50 μg/mL methylglyoxal. A solution of each analyzed compound was prepared in water at a concentration of 100 mg/mL.

Working standard solutions of the analyzed compounds were prepared in water by dilution of the solution to a higher concentration. The following concentrations were prepared: 0.01, 0.1, 0.2, 0.5, 1.0, 2.0, 4.5, 5.0, 7.5, 10.0, 20.0, 25.0, and 50.0 mg/mL for ethylene glycol, 1, 5, 15, 50, 100, and 500 μg/mL for glyoxal, and 5, 10, 15, 25, and 50 mg/mL for glycolic acid. Glyoxylic acid and oxalic acid were analyzed qualitatively at a concentration of 500 μg/mL. Calibration points and quality control (QC) samples were prepared by mixing the appropriate working solution with blank biological material. Linearity was evaluated in final concentrations of 1, 10, 20, 50, 100, 200, 450, 500, 750, 1000, 2000, 2500, and 5000 μg/mL (for ethylene glycol), 0.1, 0.5, 1.5, 5, 10, and 50 μg/mL (for glyoxal), and 0.5, 1, 1.5, 2.5, and 5 mg/mL (for glycolic acid). QC samples were prepared at three concentration levels: 0.5, 5, and 50 μg/mL (for glyoxal) and 0.5, 1.5, and 50 mg/mL (for glycolic acid), and at four concentration levels: 20, 100, 450, and 2500 μg/mL (for ethylene glycol). The latter results from the fact that the calibration curve for ethylene glycol was prepared over a wide range to meet the requirements for determining a very broad spectrum of concentrations observed in both clinical and postmortem poisoning cases.

### 2.4. Sample Preparation

**Figure 3:**
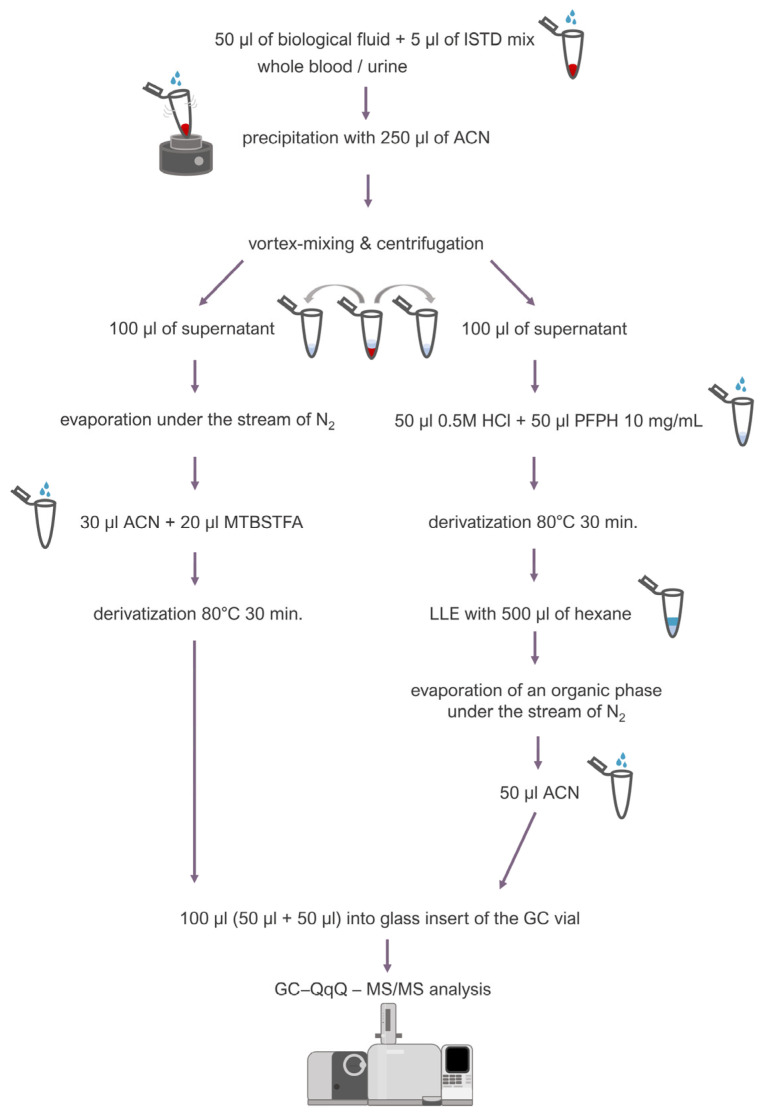
Sample preparation procedure.

Fifty microliters (50 µL) of blood or urine was transferred into an Eppendorf-type tube (2 mL), and 5 μL of an internal standard mixture (containing BHB-*d_4_* 1 mg/mL, ethylene glycol-d4 1 mg/mL, and methylglyoxal 50 μg/mL) was added. Then, 250 μL of ice-cold acetonitrile was added while vortex-mixing the sample in order to precipitate proteins. After centrifugation (13,500 rpm, room temperature), 200 μL of a supernatant was collected and transferred into two separate tubes: 100 μL into a tube (a) and another 100 μL into a tube (b). The tube (a) was placed under a stream of inert nitrogen to evaporate the supernatant, and then 30 μL of acetonitrile and 20 μL of a derivatization reagent (MTBSTFA 10 mg/mL in acetonitrile) were added to the dry residues. Derivatization was carried out for 30 min at 80 °C. To the tube (b), 100 μL of supernatant, 50 μL of 0.5 M HCl, and 50 μL of derivatization reagent (PFPH 10 mg/mL in hexane) were added, and derivatization was carried out at 80 °C for 30 min together with the tube (a). Afterwards, liquid–liquid extraction (LLE) was carried out using 500 μL of hexane. The organic phase was then evaporated under a stream of inert nitrogen, and the dry residues were dissolved in 50 μL of acetonitrile. The final step was to transfer the contents of both tubes: 50 μL from tube (a) and 50 μL from tube (b), to the glass insert of the GC vial. The described procedure is presented in Figure 3.

Because the concentrations of ethylene glycol (case 2) and glycolic acid (case 3) were above the upper limit of quantification for those compounds, ULOQ (5000 μg/mL), the assay was repeated. Blood and urine samples were diluted with water (LC-MS grade) 2-fold.

### 2.5. Instrumentation

Analyses were conducted using a gas chromatograph (GC, Shimadzu QP2010; Kyoto, Japan) operated with an autosampler AOC-20s and an autoinjector AOC-20i (Shimadzu, Milan, Italy). The separation of analytes was performed with the use of a SH-RXI-5MSi column (30 m × 0.25 mm i.d., 0.25 µm film thickness; Shimadzu, Bellefonte, PA, USA). The column temperature was initially held at 50 °C for 1 min, then increased at a 20 °C/min rate to 190 °C, then increased at a 25 °C/min rate to 220 °C, and then increased at a 35 °C/min rate to 310 °C and held there for 2 min. Helium (purity 6.0, Messer, Gumpoldskirchen, Austria) was used as a carrier gas at a flow rate of 1.6 mL/min. The syringe size was 10 µL. The injection volume was 0.2 μL. A splitless injection mode was applied with a sampling time of 1.0 min. The injector temperature was 250 °C. The syringe was set to auto cleaning by pre-injecting ethyl acetate and acetonitrile. The total run time was 13.77 min.

Detection of the analyzed compounds was achieved with the use of a triple-quadrupole mass spectrometer (QqQ, Shimadzu TQ8040, Kyoto, Japan). The spectrometer was equipped with an electron impact (EI) ion source. The determination of the analytes was carried out in the multiple reaction monitoring mode (MRM). The following MS parameters were fixed: ion source temperature of 250 °C, interface temperature of 300 °C, electron ionization energy of 70 eV, the detector voltage set at 1.0 kV. A summary of precursor and product ions, collision energies, loop time, and retention time for each compound is presented in Table 1.

### 2.6. Validation

Only compounds quantified by the method were validated, i.e., ethylene glycol, glycolic acid, and glyoxal. The developed method was validated according to the recommendations of the German Society of Toxicological and Forensic Chemistry (GTFCh) [30]. Validation parameters included selectivity, linearity, precision, accuracy, the limit of quantification, carryover, recovery, the matrix effect, and the sample dilution effect. The validation process was carried out similarly to the previous paper published by the authors [31].

#### 2.6.1. Selectivity

Ten different lots of blank whole blood or urine samples from different origin were tested for possible endogenous interference substances at the retention times of ethylene glycol, glyoxal, glycolic acid, glyoxylic acid, oxalic acid, and ISTD.

#### 2.6.2. Linearity

Linearity was evaluated by analysis of working solutions mixed with biological fluid in order to achieve final calibration concentrations of: 1, 10, 20, 50, 100, 200, 450, 500, 750, 1000, 2000, 2500, and 5000 μg/mL (for ethylene glycol), 0.1, 0.5, 1.5, 5, 10, and 50 μg/mL (for glyoxal), and 500, 1000, 1500, 2500, and 5000 μg/mL (for glycolic acid). A linear calibration model was applied, and the coefficient of determination (*R^2^*) was determined for each calibration curve. According to the acceptance criteria used, the coefficient of determination should meet the condition of: *R^2^* ≥ 0.995.

#### 2.6.3. Precision and Accuracy

The intra-day and inter-day precision and accuracy were estimated by replicating analysis (*n* = 5) of QC samples at concentration levels: 20, 100, 450, and 2500 μg/mL (for ethylene glycol), 0.5, 5, and 50 μg/mL (for glyoxal), and 500, 1500, and 5000 μg/mL (for glycolic acid). Precision was defined as the relative standard deviation (RSD%), while accuracy was expressed as the mean relative error (RE%).

#### 2.6.4. Carryover

To evaluate the carryover, three blank samples without analytes were analyzed after the highest calibration point (at the concentration of 5000 μg/mL for ethylene glycol, 50 μg/mL for glyoxal, and 5000 μg/mL for glycolic acid) for each substance. The blood or urine samples containing internal standards without determined analytes were evaluated for the presence of ethylene glycol, glyoxal, and glycolic acid.

#### 2.6.5. The Limit of Detection and Limit of Quantification

The lower limit of quantification (LLOQ) was interpreted as the concentration at which the relative standard deviation (RSD%) and relative error (RE%) do not exceed 20% and 15%, respectively. The lower limit of detection (LLOD) was calculated from the signal-to-noise ratio, assuming that the ratio should equal 3.

#### 2.6.6. Recovery and Matrix Effect

The recovery and matrix effect were evaluated for each analyte at concentration levels: 20, 100, 450, and 2500 μg/mL (for ethylene glycol), 0.5, 5, and 50 μg/mL (for glyoxal), and 500, 1500, and 5000 mg/mL (for glycolic acid). The recovery [32] and matrix effect [33] were determined with the use of the equations presented below (expressed in percent, *n* = 5).

$$Recovery\;[\%]=\left(\frac{{Response}_{post-extracted\;spiked\;sample}}{{Response}_{pre-extracted\;spiked\;sample}}\right)\times 100$$

$$Matrix\;effect\left[\%\right]=\left(\frac{{Response}_{post-extracted\;spiked\;sample}}{{Response}_{non-extracted\;neat\;\;sample}}-1\right)\times 100$$

#### 2.6.7. Dilution Effect

The blood and urine samples (*n* = 5) were spiked with ethylene glycol, glyoxal, or glycolic acid working solution to obtain the final concentrations of 2500, 50, and 5000 μg/mL, respectively. Next, the samples were diluted 10-fold with water, and then the precision and accuracy were determined. The unacceptable dilution effect was established when RSD% and RE% exceeded 20% and 15%, respectively.

## 3. Results

### 3.1. Chromatographic Separation and Optimization of Mass Spectrometer Parameters

Optimization of chromatographic and mass spectrometry conditions was achieved by analyzing solutions of ethylene glycol, ethylene glycol-d_4_, glycolic acid, glyoxylic acid, oxalic acid, and BHB-d_4_ at a concentration of 500 μg/mL, as well as glyoxal and methylglyoxal solutions at a concentration of 5 μg/mL each.

**Figure 4:**
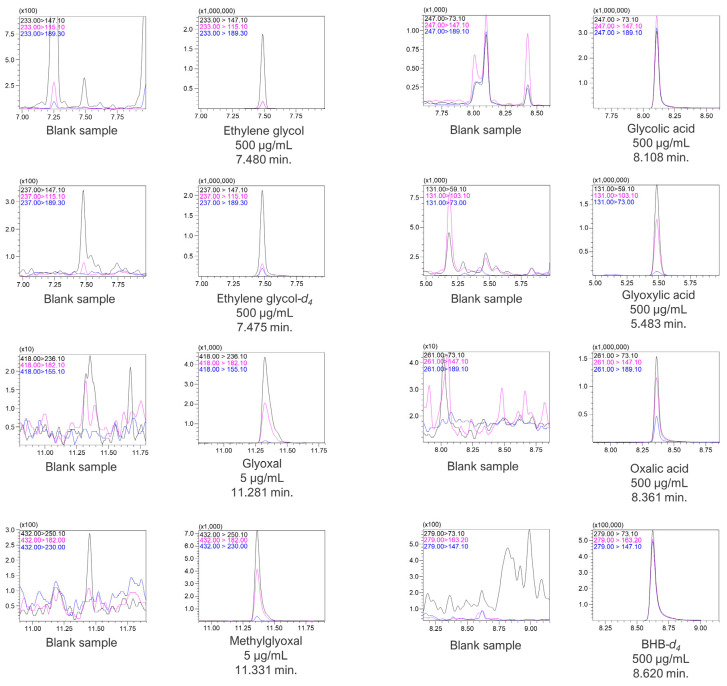
Chromatograms with MRM transitions and retention times of the analytes and ISTD.

The analytes were analyzed under the abovementioned chromatographic conditions in the scan mode in the range of 40–500 *m/z*. Chromatographic signals for all compounds were acquired with the corresponding mass spectrometry spectra. Glyoxal and methylglyoxal PFPH derivatives exhibited precursor ions of 418 *m/z* and 432 *m/z*, respectively. However, for MTBSTFA derivatives, i.e., for ethylene glycol, ethylene glycol-d_4_, glycolic acid, glyoxylic acid, oxalic acid, and BHB-d_4_, no molecular ions were observed. Instead, ions with *m/z* values lower by 57 than the molecular weight for each compound were present on the MS spectra. The molecular ions (in the case of glyoxal and methylglyoxal) as well as [M-57]^+^ ions (for the rest of the compounds) of all analytes and internal standards were fragmented by collision gas in a collision cell, and the products were analyzed in the scan mode of Q3. The MS scan spectra, as well as the product ion scan spectra for all compounds, are presented in Supplementary Material S2. The most optimal collision energies (CE) were selected with the use of the MRM method optimization software. Chromatograms with selected MRM transitions for each analyte as well as for internal standards are presented in Figure 4.

### 3.2. Validation Process

Very good validation parameters were achieved in the developed method. No interfering ion current signals were observed at the retention times of the analytes or internal standards. Taking into consideration the calibration curve on blood, the value of the coefficient of determination (R^2^) was 0.9992 and 0.9999 for glyoxal and glycolic acid, respectively. As it turned out, ethylene glycol exhibited two linear ranges (i.e., 1–450 μg/mL and 450–5000 μg/mL), which we decided to incorporate into this study since the observed concentrations of this compound in poisoning cases are very broad. Hence, for the first linear range, the R^2^ parameter was 0.9996, and for the second, it was 0.9960. The lower limit of quantification (LLOQ) was 1.0 μg/mL for ethylene glycol, 0.1 μg/mL for glyoxal, and 500 μg/mL for glycolic acid. The quantified limit of detection (LOD) was 14.2 ng/mL, 2.7 ng/mL, and 623.4 ng/mL for ethylene glycol, glyoxal, and glycolic acid, respectively. Intraday precision and accuracy were established at the previously given QC concentration levels for all three analytes in whole blood. Intraday precision did not exceed 6.8, 12.1, and 10.6 (RSD%) for ethylene glycol, glyoxal, and glycolic acid, respectively. Accuracy values did not exceed 4.7, −8.0, and −6.7 (RE%) for ethylene glycol, glyoxal, and glycolic acid, respectively. Interday precision for ethylene glycol, glyoxal, and glycolic acid did not exceed 7.8, 7.5, and 11.1 (RSD%), and interday accuracy for those compounds did not exceed 9.4 (ethylene glycol), −4.0 (glyoxal), or 11.3 (glycolic acid). Recovery and matrix effect values were in the range of 96.8–101.9% and −3.2–1.9% (ethylene glycol), 101.5–107.3% and 1.3–7.4% (glyoxal), 84.9–88.0% and −15.1–−12.0 (glycolic acid). A negative matrix effect was observed for ethylene glycol and glycolic acid, but not for glyoxal. This may be due to several factors, including the fact that the high polarity of the diol and carboxylic acid can lead to strong interactions with residual water and other polar components in the sample matrix, which can enhance ion suppression. Another factor might arise from the fact that those compounds can experience significant ion suppression if co-extracted matrix components co-elute with them and compete for ionization in the mass spectrometer [32]. Furthermore, there were no substances carried over between samples. In the case of forensic toxicology, it is possible for the samples to have concentrations above the upper limit of quantification of the method (especially in fatal outcomes) [34,35]. In such cases (similarly to those described in this paper), the dilution effect parameter provides additional information about method suitability. The effect of dilution for samples of 5000 μg/mL (ethylene glycol), 50 μg/mL (glyoxal), and 5000 μg/mL (glycolic acid) expressed as RE did not exceed ±15%. Thus, it can be concluded that a 10-fold dilution of the real samples did not significantly affect the result of the analysis of the determined compounds. A summary of the validation results is presented in Table 2.

In the case of urine matrix precision, values were in the rage of 1.6–11.6% RSD. Accuracy values did not exceed 8%. Recovery values were in the range of 97.1–103.3%. Details of the validation performance are presented in Supplementary Material Table S3.

### 3.3. Application of the Method to Authentic Cases

The developed GC–QqQ–MS/MS method was applied for the determination of ethylene glycol, glyoxal, and glycolic acid in three authentic cases where whole blood and urine were collected during autopsy. What is more, glyoxylic acid and oxalic acid were meant to be detected qualitatively. Toxicological analysis of authentic materials revealed the following concentrations of the analytes: ethylene glycol was in a range of 61–5204 μg/mL and 201–6678 μg/mL in whole blood and urine, respectively; glycolic acid was in a range of 1780–5901 μg/mL in whole blood and 2564–5801 μg/mL in urine samples. Glyoxal was determined in both biological materials only in case 1, where its concentrations were 4.52 μg/mL and 1.59 μg/mL in blood and urine, respectively. In case 3, glyoxal was present only in urine at a concentration of 0.31 μg/mL. Glyoxylic and oxalic acids were also detected in most of the materials. Only in the urine sample from case 3, oxalic acid was not present. The detailed results concerning the analysis of authentic samples are presented in Table 3.

## 4. Discussion

One of the most frequently used methods of determining ethylene glycol in biological samples is gas chromatography coupled with mass spectrometry (GC–MS). Thus, the authors focused on discussing the issue of determining ethylene glycol in biological samples in the context of the only GC–MS methods developed to date. The summary of papers concerning gas chromatography–mass spectrometry methods used to determine ethylene glycol is presented in Table 4.

### 4.1. Optimization of Sample Preparation

The analysis of postmortem biological material is very often accompanied by the problem of the small amount of sample that can be collected during the autopsy. For example, decease is linked to the relaxation of the sphincter muscles, resulting in the excretion of urine, so the amount of urine present in the urinary bladder may be very small. However, urine is an important material in toxicological analysis, as metabolites of drugs, xenobiotics, and endogenous substances are excreted mainly through urine. In the case of charred cadavers, exsanguinated cadavers, or newborns, there may be a problem in collecting a sufficient amount of blood for subsequent testing. In the context of toxicological analysis of human postmortem samples, it is often challenging to obtain a large volume of biological material. This poses two main issues: first, in some cases, only a small amount of blood is available for testing (such as in accidents involving significant blood loss or cases involving neonates) [36], and second, several or a dozen different tests need to be performed from a single vial of blood, making it necessary to use methods that require a small amount of material for testing. The above-described situations elucidate the need to use as little biological material as possible in toxicological analysis while simultaneously providing good sensitivity and selectivity. A review of the available literature for the analysis of ethylene glycol by GC–MS showed that 50 μL is one of the smallest volumes of biological material used to date, which was also used in six other papers [14,16,37,38,39,40]. Other authors used 100 μL [15,41], 200 μL [42,43], and 250 μL [44] of biological material in their analyses. Only one author [17] used a smaller amount of biological material, i.e., 20 μL. Using as little as 50 μL of a biological sample is not only important in the more specific cases described above but also in routine toxicological analysis since it includes performing many additional analyses of other pharmaceutical drugs, illicit drugs, volatile substances, etc. Thus, the more sensitive the method is and the less material is used, the more possibilities there are for conducting other analyses, which in consequence leads to significant improvements in the toxicological expertise possibilities and determining the cause of death.

Another important factor in the quantitative analysis of postmortem biological material is the selection of an appropriate internal standard. Authors determining ethylene glycol by GC–MS used substances such as 3-(4-chloro-phenyl)propionic acid [15]. However, the most commonly used internal standard was 1,3-propanediol [14,15,16,17,38,41,42,43] or butanediol isomers [39,40]. Although 1,3-propanediol exhibits similar physical and chemical properties to the analytes, this compound cannot be used in the analysis of postmortem biological material as it is formed endogenously by the microbial transformation of glycerol [45]. During the development of a method, it must be taken into consideration that using compounds that are formed endogenously as internal standards leads to the falsification of the obtained quantitative analysis results. Therefore, a substance that is potentially already present in postmortem or antemortem biological material cannot be used as an internal standard. The solution is to use deuterated derivatives of the analyzed substances. The internal standards used in the developed method, as well as those used by Wurita et al. [44] and Robson et al. [37], were deuterated derivatives of the analytes. In the presented method, ethylene glycol-*d_4_* served as the ISTD for ethylene glycol, and BHB-*d_4_* was used for the determination of glycolic acid, as those compounds belong to the hydroxy carboxylic acid group. Such an approach provided chemical as well as physical similarity of internal standards to the analytes. In addition, it made quantitative analysis independent of the possibility of postmortem endogenous chemical compounds presence.

Methylglyoxal was the only compound used as an internal standard that did not contain any deuterium atoms. However, the endogenous concentration of methylglyoxal in human plasma is maintained at a low level (3.6–72 ng/mL [46,47,48]), significantly lower than the amount present in a biological sample as an internal standard (5 μg/mL), which does not have an impact on the final results of the glyoxal determination.

**Table 4:** Comparison of GC–MS methods used for the analysis of ethylene glycol in biological material.

Reference	Analysed Substances	Analysed Biological Material	Matrix used for Calibration	Sample Volume [μl]	ISTD	Derivatization Agent	Sample Preparation Technique	LOQ [μg/mL]	Injection Volume [μL]	MS Mode
[44]	ethylene glycol	whole blood	whole blood	250	ethylene glycol-*d_6_*	heptafluorobutyric anhydride	LLE with *n*-hexane	nd	1.0	SIM
[42]	ethylene glycol	plasma urine	plasma	200	1,3-propanediol	pivalic anhydride /triethylamine /methanol (20:1:1 *v*/*v*/*v*)	precipitation with acetone	100	1.0	scan
[37]	ethylene glycol	plasma	water	50	ethylene glycol-*d_4_*	dimethylformamide + *N*,*O*-bis-(trimethylsilyl)-trifluoroacetamide	–	50	–	SIM
[14]	ethylene glycol glycolic acid	serum	serum	50	1,3-propanediol	*N*-(*tert*-butyl-dimethylsilyl)-*N*-methyltrifluoroacetamide	precipitation with acetic acid and ACN	10	1.0	MRM
[15]	ethylene glycol glycolic acid	serum urine	serum	100	1,3-propanediol 3-(4-chloro-phenyl)propionic acid	isobutyl chloroformate	extraction with borate buffer in pH 9	50	1.0	MRM
[16]	ethylene glycol glycolic acid	whole blood	whole blood	50	1,3-propanediol	*N*-(*tert*-butyl-dimethylsilyl)-*N*-methyltrifluoroacetamide	precipitation with ACN	50	1.0	scan
[41]	ethylene glycol	serum	bovine serum	100	1,3-propanediol	phenylboronic acid	precipitation with ACN	nd	0.5–1.0	–
[38]	ethylene glycol glycolic acid	plasma urine	plasma	50	1,3-propanediol	*N*,*O*-bis-(trimethylsilyl)-trifluoroacetamide	nd	50	1.0	MRM
[39]	ethylene glycol	serum	serum	50	1,2-butanediol	4-carboethoxy-hexafluorobutyryl chloride	LLE with acetone	nd	2.0	scan
[40]	ethylene glycol	serum	serum	50	1,4-butanediol	pentafluorooctanoyl chloride	LLE with acetone	nd	1.0–2.0	scan
[43]	ethylene glycol	plasma	plasma	200	1,3-propanediol	pivalic anhydride /triethylamine /methanol (20:1:1 *v/v/v*)	LLE with acetone	nd	0.5	scan
[17]	ethylene glycol glycolic acid	serum plasma urine	bovine serum albumin	20	1,3-propanediol	bis- *N*,*O*-trimethylsilyl trifluoroacetamide	–	0.39	1.0	SIM
Presented method	ethylene glycol glycolic acid glyoxal glyoxylic acid ^a^ oxalic acid ^a^	whole blood urine	whole blood and urine	50	ethylene glycol-*d_4_* BHB-*d_4_* methylglyoxal	*N*-tert-butyldimethylsilyl-*N*-methyltrifluoroacetamide + *tert*-butyldimethylchlorosilane, pentafluorophenylhydrazine	precipitation with ACN, LLE with hexane	1.0	0.2	MRM

– not specified; ISTD—internal standard; LOQ—lowest limit of quantification for ethylene glycol; LLE—liquid–liquid extraction; nd—no data available; ACN—acetonitrile; MRM—multiple reaction monitoring; SIM—selected ion monitoring; ^a^—analyzed qualitatively.

### 4.2. Optimization of Derivatization

Given that a necessary step for the determination of ethylene glycol by gas chromatography is its derivatization, many different methods have been developed. Single papers described the use of derivatization reagents such as isobutyl chloroformate [15] or phenylboronic acid [41]. Maurer et al., in their two papers [42,43], described the use of pivalic anhydride for the determination of ethylene glycol and diethylene glycol. Other reagents often used were various types of organic halogens: heptafluorobutyric anhydride [44], 4-carboethoxyhexafluorobutyryl chloride [39], or pentafluorooctanoyl chloride [40]. The most commonly used derivatization reagents, however, were compounds that gave methylsilyl derivatives of the analytes. As in the work described here, Porter et al. [14] and Rosano et al. [16] used N-(tert-butyl-dimethylsilyl)-N-methyltrifluoroacetamide. Three other papers chose a similar reagent for derivatization: N,O-bis-(trimethylsilyl)-trifluoroacetamide [17,37,38], differing from the former in the alkyl moiety attached to the silicon atom. All three mentioned derivatives can be successfully determined by GC–MS; however, the derivative with the tert-butyl moiety has a greater mass, which is an advantage from the point of view of MS analysis (because it provides better selectivity and sensitivity of the method).

However, it should be noted that none of the aforementioned studies have addressed the analysis of all ethylene glycol metabolites, and therefore, they did not analyze compounds with other functional groups than the hydroxyl group, which can be derivatized using the aforementioned reagents. In this study, however, the authors developed a comprehensive method for the simultaneous determination of both alcohols and carboxylic acids, as well as aldehydes, from a single biological sample. This enabled the use of two derivatizing agents, namely N-tert-butyldimethylsilyl-N-methyltrifluoroacetamide with 1% tert-butyldimethylchlorosilane (MTBSTFA) for derivatization of hydroxyl groups in alcohols and carboxylic acids and pentafluorophenylhydrazine (PFPH) for derivatization of the ketone group. The positive results of such a comprehensive approach, along with the simplicity of sample preparation, demonstrate that compounds with various functional groups can be simultaneously determined from a single biological sample, requiring different derivatization reactions. Moreover, the use of the two described reagents allows for the extension of the method to compounds from the amine group (derivatized by MTBSTFA) or aldehydes (derivatized by PFPH), while maintaining the entire procedure, which may also prove useful in toxicological diagnostics. Moreover, the stability of the different derivatives obtained, which are combined with each other in the final stage of sample preparation, was checked with respect to the individual derivatives of the standards of the analyzed substances. It follows that combining solutions of TBDMS and PFPH derivatives with each other has no negative effect on the stability of these compounds.

### 4.3. Optimization of GC–MS Analysis

The developed method enabled simultaneous analysis of all analytes, i.e., ethylene glycol and its metabolites. The use of multiple reaction monitoring (MRM) mode provided good selectivity by monitoring a series of transitions of selected precursor ions to their selected fragment ions. In forensic toxicology, where it is important to provide well-grounded expertise based on the results of the analyses, it is especially important to obtain reliable identification of the analyte. In the analyses of ethylene glycol in biological material by GC–MS described in the literature, MRM mode was used by three authors [14,15,38], while three papers included selected ion monitoring (SIM) mode [17,37,44]. However, some authors used scan mode [16,39,40,42,43] in their analyses, which is a far less sensitive mode compared to MRM. Thus, more biological material and higher injection volumes are required in order to obtain satisfying limits of quantification for the analyzed compounds. This, on the other hand, can result in an enlarged matrix effect involving the presence of more matrix ions in the MS detector, which leads to the competition of these ions with analyte ions. In consequence, it increases noise, decreases the signal-to-noise ratio of compounds of interest, and negatively affects the detection process.

In the developed method, the limits of quantification were 1 μg/mL, 0.1 μg/mL, and 500 μg/mL for ethylene glycol, glyoxal, and glycolic acid, respectively. Reported postmortem concentrations of ethylene glycol in blood range from 152 μg/mL [49] to 7.8 mg/mL [50], while in urine they range from 600 μg/mL to 10.8 mg/mL [51]. This means that the developed method is applicable in the analysis of lethal ethylene glycol poisoning, which is also confirmed by the upper limits of the calibration curves and the wide range of concentrations analyzed for ethylene glycol after taking into account the two ranges of linearity of the calibration curves. Limits of quantification of ethylene glycol in biological material (blood, plasma, serum, urine) by GC–MS achieved by other authors are mainly 50 μg/mL [15,16,37,38]. Maurer et al. [42] defined the LOQ as 100 μg/mL, while Porter et al. [14] developed a method with an LOQ of 10 μg/mL. Two papers described a limit of quantification lower than in the presented work. In the paper by Wurita et al. [44], the calibration curve for ethylene glycol is in the range of 0.4–400 ng/mL. The authors did not provide the exact values for LOQ, as they used blood from healthy patients, which they claimed already contained ethylene glycol. However, it is worth mentioning that in this case, the quantification was based only on establishing the signal-to-noise ratio for one ion with a specific *m/z* value (selected ion monitoring, SIM). What is more, the authors did not examine precision or accuracy to check if the concentrations meeting the specified signal-to-noise ratio values were also characterized by expected RE and RSD values. Vanhee et al. [17] also presented a sensitive method for the determination of, among others, ethylene glycol and glycolic acid in their study. The limit of quantification (LOQ) values reported by these authors are 1.19 and 0.39 µg/mL for glycolic acid and ethylene glycol, respectively. However, as the aforementioned authors declare, these are theoretical values calculated from the signal-to-noise ratio. It is possible that those values may deviate from the real ones, if one takes into account, for example, the effect of the matrix on analyte signals at low concentrations. Compared to this, the LOQ values presented in this work are higher but are determined experimentally, as the lowest concentration tested was determined from the concentrations observed in poisoning cases, whose RSD% and RE% values matched the validation assumptions (shown in Table 2). However, if the theoretically calculated LOQ values in this work were taken into account, they would be significantly lower (on the order of 0.6 μg/mL for ethylene glycol, 1.5 μg/mL for glycolic acid, and 0.01 μg/mL for glyoxal) making this method the most sensitive in terms of determining both ethylene glycol and glycolic acid. What is more, several issues need to be addressed that would preclude the application of Vanhee et al. [17] method in postmortem forensic toxicology cases. By investigating serum tissue, which serves as a much purer biological matrix than whole blood, the authors were able to employ a significantly larger injection volume for analysis, as it did not result in as significant matrix effects and competition of its ions with the analyte ions. Consequently, they could also utilize a smaller volume of biological material, which is an undeniable advantage of the method. However, for forensic toxicology purposes in postmortem samples, blood, which constitutes a much more complex biological matrix, is the most commonly secured biological material. Furthermore, the selection of the internal standard, namely 1,3-propanediol, would not be suitable for postmortem samples because, as was previously mentioned, this compound is formed endogenously. Therefore, although the method proposed by Vanhee et al. [17] is exceedingly sensitive and utilizes a small volume of material, it would not be applicable for the analysis of postmortem blood samples.

### 4.4. Fragmentation Pathway of the Analytes

When analyzing the MS spectra collected in product ion scan mode (all spectra are available in Supplementary Material S2), characteristic product ions for each type of derivative are present. For glyoxal and methylglyoxal, compounds that were derivatized with the use of pentafluorophenylhydrazine, bis-PFPH derivatives were formed with M+: 418 *m/z* and 432 *m/z* for glyoxal and methylglyoxal, respectively. Except for the precursor ions, one characteristic product ion for each analyte was present (236 *m/z* for glyoxal and 250 *m/z* for methylglyoxal), with the *m/z* value dependent on the alkyl chain structure of the compound of interest. Those characteristic ions are formed through the cleavage of the double bond between two nitrogen atoms in one of the hydrazine moiety. On the other hand, product ions with *m/z* values of 209 and 182 should be expected in the spectra of all PFPH derivatives as they come from the derivatization agent itself (182 *m/z*) or contain additional carbon atoms (209 *m/z*).

For TBDMS derivatives, i.e., ethylene glycol, ethylene glycol-d_4_, glycolic acid, glyoxylic acid, oxalic acid, and BHB-d_4_, a more complex fragmentation pathway could be considered. Product ions: 189 *m/z*, 147 *m/z,* and 73 *m/z* are expected for all bis-TBDMS derivatives, except for glyoxylic acid, as they are formed upon detachment from the cyclic molecule fragment containing the tert-butyldimethylsilyl moiety, oxygen from the hydroxyl group, and the silicon atom along with the methyl groups constituting part of the second tert-butyldimethylsilyl moiety. No molecular ions are observed for bis-TBDMS derivatives; however, characteristic ones are present for each bis-TBDMS derivative, depending on their structure. The fragmentation of such compounds, described by Harvey et al. [52], occurs through the loss of a tert-butyl radical from a tert-butyldimethylsilyl group, and a cation is formed, which gives an intense peak on the MS spectrum with *m/z* value lower by 57 Da than that of the expected molecular ion. In addition, in the case of diols, Harvey et al. described a mechanism for cyclization of the resulting [M–57]^+^ ion.

The abovementioned mechanism of fragmentation relates only to bis-TBDMS derivatives. In the case of compounds with only one hydroxy group, such as glyoxylic acid, the loss of a tert-butyl radical also occurs (resulting in 131 *m/z* ion), but no cyclization is possible because of the lack of a single-bounded oxygen, as glyoxylic acid has a carbonyl group with double-bounded oxygen. For this reason, the fragmentation pattern of this compound differs from that of bis-TBDMS derivatives, resulting in the absence of characteristic fragments with *m/z* 189, 147, or 73. Furthermore, in the case of glyoxylic acid-TMBDS, with a monoisotopic mass of 188.08687 Da, the expected molecular ion would be 188 *m/z*. However, in the MS spectrum obtained in scan mode, an ion at 189 *m/z* is observed. This may be attributed to the fact that oxygen from carboxylic group has the ability to transition from a ketone to an enol form. In polar solvents, the preferred form of glyoxylic acid is not the ketone but the enol, whose monoisotopic mass with the attached TBDMS group is 189.094247 Da, hence coinciding with the ion observed in the scan spectrum. Considering that sample preparation for derivatization using TBDMS involved the use of acetonitrile as a solvent, which is a polar solvent, it can be assumed that the formation of the enol form is expected. Additionally, this is supported by the property of acetonitrile, which can favor the stabilization of the enol through dipole interactions. It is worth noting that the formation of the enol would not lead to the attachment of a second TBDMS group during derivatization, as enolization can only occur at the oxygen of the previous carboxylic group. Two TBDMS groups positioned so closely are not feasible due to the large size of this group.

### 4.5. Analysis of Biological Material

The developed analytical method allowed the determination of ethylene glycol and its metabolites in all postmortem samples that were examined. Based on ethylene glycol concentration values in fatal and non-fatal cases presented in Section 1.1. Epidemiology of ethylene glycol intoxications, it can be concluded that the developed method could be successfully used in postmortem biological sample examination. The validation process, which exhibited parameters in accordance with forensic toxicology analysis requirements, proves the usefulness of the method in toxicological analysis for judicial authorities. In forensic toxicology, plasma or serum is seldom available for analysis because of the decomposition of biological materials and blood hemolysis. Therefore, it is crucial to have a precise, sensitive, and accurate method for detecting xenobiotics, specifically in the toxicological examination of postmortem biological fluids [53]. What is more, it should be taken into consideration that the described method could possibly be implemented in the clinical diagnostic process of ethylene glycol intoxication.

Last but not least, in case 1, the concentration of ethylene glycol is remarkably lower than in other cases. Taking into consideration previously developed GC–MS methods, in some of them the concentration determined in blood (60.87 μg/mL) is below or close to the LOQ of the method. Thus, there is a possibility that the compound might not be determined, which leads to problems with ethylene glycol intoxication diagnosis. Developing a method that enables the determination of ethylene glycol metabolites, especially glycolic acid (whose synthesis is a limiting step in ethylene glycol metabolism), makes it possible to detect the potential uptake of a xenobiotic even when the initial compound is absent or its concentration is very low. The presence of other metabolites, i.e., glyoxal, glyoxylic acid, and oxalic acid, additionally confirms the ethylene glycol intoxication diagnosis. According to a literature review of both ethylene glycol determination methods and poisoning cases, in no case was the concentration of glyoxal determined in biological material. The method developed and described in this work allows quantitative determination of this compound and provides additional interpretative possibilities, which is an undeniable advantage.

Two compounds that are metabolites of ethylene glycol were analyzed only qualitatively. Glyoxylic acid is an intermediate metabolite in the detoxification of ethylene glycol. Although glyoxylic acid is an intermediate metabolite, its quantification does not provide significant additional clinical information that could not be obtained by measuring other more stable and clinically relevant metabolites, such as glycolic acid. In addition, glyoxylic acid may occur in low concentrations naturally in the body as a byproduct of various metabolic processes [54]. Therefore, its presence in small amounts does not necessarily indicate ethylene glycol poisoning, further complicating the interpretation of quantitative results. As for oxalic acid, on the other hand, it is the end product of the metabolism of many compounds, including ethylene glycol, but also other dietary sources and endogenous metabolic processes [55]. Hence, the mere presence of oxalic acid in the blood is not specific to ethylene glycol poisoning, as it can also be formed from other sources. The determination of its concentration does not necessarily provide specific diagnostic information related to poisoning. A much better diagnostic parameter, which is often described in poisoning cases [56,57,58,59,60,61,62,63,64,65,66,67,68,69,70,71,72,73,74,75,76,77,78,79,80,81,82,83,84,85,86,87,88], is the presence of calcium oxalate crystals in the kidneys and other organs.

## 5. Conclusions

This study demonstrates the successful application of simultaneous derivatization of various functional groups for the determination of compounds belonging to alcohols, carboxylic acids, and aldehydes. This was illustrated through the example of ethylene glycol and its metabolite determination in postmortem biological material; however, the developed method also provides opportunities for its application in preparing samples for a wide range of chemical substance investigations, encompassing various functional groups, while concurrently conserving sample quantity. A major advantage of the method is the very small volume of biological sample needed for analysis (50 μL), which is particularly important for the study of postmortem material. The possibility of simultaneous determination of ethylene glycol and its metabolites greatly increases the interpretative possibilities in the field of forensic toxicology. Confirmation of the ability to determine ethylene glycol, glyoxal, and glycolic acid in such a complex matrix as a postmortem sample was provided by the analysis of six samples from authentic poisoning cases. In addition, it can be concluded that the developed method could also be used in the clinical diagnosis of ethylene glycol poisoning.

### Novelty of the Method

- Simultaneous derivatization of alcohol’s, aldehyde’s, and carboxylic acid’s analogs from one sample.
- The method allows simultaneous ethylene glycol and its metabolite determination within a short time.
- Biological sample volume is reduced to only 50 μL.
- Method was successfully applied to three authentic postmortem cases of intoxication where concentrations of ethylene glycol and its metabolites were presented, allowing for an increase in toxicological knowledge on the subject.
- The lowest LOQ value (1 μg/mL) for ethylene glycol was achieved.

## Figures and Tables

**Table 1:** Multiple reaction monitoring (MRM) conditions used in the GC–QqQ–MS/MS method for quantification of ethylene glycol and its metabolites in biological samples.

Substance	Molecular Weight [g/mol]	Retention Time [min]	Theoretical M^+^ value [Da]	Precursor ion [*m/z*]	Product ion [*m/z*]	SRM Ratio [%] ^a^	Loop Time [sec]	Collision Energy [V]
Ethylene glycol	62.07	7.480	304.224834	233.0	147.1 *	100.00	0.22	8.0
					115.1	10.25		8.0
					189.3	10.30		2.0
Glyoxal	58.04	11.281	418.02708	418.0	236.1 *	100.00	0.22	11.0
					182.1	40.79		29.0
					155.1	4.34		35.0
Glycolic acid	76.05	8.108	304.188448	247.0	73.1 *	100.00	0.22	29.0
					147.1	119.92		20.0
					189.1	102.04		8.0
Glyoxylic acid	74.04	5.482	188.086322	131.0	59.1 *	100.00	0.22	10.0
					103.1	55.20		10.0
					73.0	5.74		15.0
Oxalic acid	90.03	8.361	318.167713	261.0	73.1 *	100.00	0.22	17.0
					147.1	96.47		8.0
					189.1	23.34		5.0
Methylglyoxal	72.06	11.331	432.04273	432.0	250.1 *	100.00	0.22	8.0
					182.0	73.36		29.0
					230.0	5.07		11.0
Ethylene glycol-*d_4_*	66.09	7.475	308.249941	237.0	147.1 *	100.00	0.22	8.0
					115.1	27.47		8.0
					189.3	8.60		2.0
BHB-*d_4_*	108.10	8.620	336.244855	279.0	73.1 *	100.00	0.22	26.0
					163.2	88.72		8.0
					147.1	87.92		20.0

BHB—3-hydroxybutanoic acid. * ions selected for quantitative analysis. ^a^—analyzed qualitatively.

**Table 2:** Parameters of the method for quantification of ethylene glycol and its metabolites in blood samples.

Substance	Calibration Curve						
	The Linear Concentration Range [μg/mL]		Internal Standard		The Coefficient of Determination (R^2^)		LLOQ [μg/mL]
Ethylene glycol	1–450		Ethylene glycol-*d*_4_		0.9996		1.0
	450–5000				0.9960		
Glyoxal	0.1–50		Methylglyoxal		0.9992		0.1
Glycolic acid	500–5000		BHB-*d*_4_		0.9999		500
**Validation Parameters**							
**Concentration Level** **[μg/mL]**	**Intraday**		**Interday**		Recovery [%] *	Matrix Effect [%] *	**Dilution Effect** RE [%] *
	Precision RSD [%] *	Accuracy RE [%] *	Precision RSD [%] *	Accuracy RE [%] *			
20 100 450 2500	2.9 0.7 6.8 4.2	−4.0 −3.2 4.7 −1.8	2.3 1.6 7.0 7.8	−6.8 −2.1 4.6 9.4	98.2 101.9 99.7 96.8	−1.8 1.9 0.3 −3.2	– – – 11.6
0.5	12.1	−8.0	7.5	−4.0	101.5	1.3	–
5	7.4	2.1	7.2	−3.2	107.3	7.4	–
50	5.8	−2.5	2.5	−1.6	102.2	2.8	−9.3
500	8.1	−6.7	7.5	11.3	84.9	−15.1	–
1500	7.3	0.4	11.1	−9.3	87.3	−12.7	–
5000	10.6	−5.9	4.7	−3.2	88.0	−12.0	−7.2

BHB—3-hydroxybutanoic acid; LLOQ—lowest limit of quantification; RSD—relative standard deviation; RE—relative error; S/N—signal-to-noise ratio. * (*n* = 5).

**Table 3:** Concentration of ethylene glycol and its metabolites in authentic postmortem biological material.

Case No.	Material	Ethylene Glycol [μg/mL]	Glyoxal [μg/mL]	Glycolic Acid [μg/mL]	Glyoxylic Acid [μg/mL]	Oxalic Acid [μg/mL]
1	whole blood	61	4.52	1780	+	+
	urine	212	1.59	2570	+	+
2	whole blood	5204	nd	1857	+	+
	urine	6678	nd	3898	+	+
3	whole blood	1008	nd	5901	+	+
	urine	1468	0.31	5801	+	nd

nd—not detected; +—detected qualitatively.

## Data Availability

The authors confirm that the data supporting the findings of this study are available within the article and its Supplementary Materials.

## Supplementary Materials

The following supporting information can be downloaded at: https://www.mdpi.com/article/10.3390/jox14030065/s1, S1. Epidemiology of ethylene glycol intoxications [18,49,50,56,57,58,59,60,61,62,63,64,65,66,67,68,69,70,71,72,73,74,75,76,77,78,79,80,81,82,83,84,85,86,87,88,89,90,91,92,93,94,95,96,97,98,99,100,101,102,103,104,105,106,107,108,109,110,111,112,113,114,115,116,117,118,119,120,121,122]; Table S1. Cases of ethylene glycol intoxications reported in the literature. S2. Mass spectrometry spectra acquired in scan mode and product ion scan mode; Table S3. Parameters of the method for quantification of ethylene glycol and its metabolites in urine samples.
