# Supplementary material for: Novel Technique for Simultaneous Ethylene Glycol and Its Metabolites Determination in Human Whole Blood and Urine Samples Using GC–QqQ–MS/MS
Source: J Xenobiot. 2024 Aug 27;14(3):1143–64. doi: 10.3390/jox14030065 (PMC11417905; doi:10.3390/jox14030065)

S1. Epidemiology of ethylene glycol intoxications

Based on the collected data, it can be stated that most of the described cases of ethylene glycol intoxication were not fatal. One-third of the analysed cases resulted in death [18,49,50,56,60,64,65,68,71,73,85,89,90-92], while 71% involved intoxications of people who were successfully treated in a hospital ward and discharged home. Among the intoxicated people, the vast majority (79%) were men; cases of poisoning of women accounted for 17 of the 81 total cases (21%). This is somewhat of a surprise given that almost one-third of the cases (29%) were confirmed suicides or suicide attempts [56,57,60–64,72,76–79,80,84,92–99], and women are more prone than men to choose poisoning as a method of a suicide [100].

9 out of 81 cases, which accounts for 11%, were described as accidental poisonings [56,64,66,74,90,101–103], while in two cases the incident was considered a homicide during the investigation [49,104]]. The most prevalent way of intoxication was the ingestion of an antifreeze product containing ethylene glycol in various concentrations [49,57,60,61,70,72,74,76–81,85,90,92–96,103–108]. Such cases accounted for 38% of the total, and among them, 13 out of 81 cases were confirmed suicide attempts. The next most common way in which poisoning occurred was the ingestion of a pure solution of ethylene glycol in various concentrations and amounts [56,57,62–64,66,73,83,85,91,99,101,102,109]. Such cases accounted for a total of 20% of all 81 cases examined. In three cases, representing 4%, poisoning occurred through ingestion of a brake fluid containing ethylene glycol. In some cases ethylene glycol poisoning was associated with alcohol consumption, including aniseed-flavoured alcohol and homemade one, as well as one case of a simultaneous ethylene glycol and methanol intoxication. One reported fatality, described as accidental poisoning, involved ingestion of a hand sanitizer [56,89,90,97,98,110,111].

In the vast majority of cases (74%), other substances detected in the biological material during the toxicological examination were not mentioned. This may be due to the non-presence of them or the fact that such analyses were not carried out. In 26% of cases, the presence of other chemical compounds was demonstrated: in 12 cases it was ethyl alcohol [18, 56, 61,71,72,76,85,93,95,96,112], in one fatal case - propylene glycol [49]. In addition to these compounds, cannabinoids [58,78] and benzodiazepines [72,78,113] were also detected. It is also noteworthy that in 17% of cases the information about suffering from depression was reported [56,62,63,70,72,76,84,90,95,96,114]. 10 out of 14 people in whom depressive disorders were described had poisoned themselves as a result of a suicide attempt, which can prove that suffering from depression is correlated with suicidal tendencies [100]. Among the cases of confirmed suicide and suicide attempts examined (24 cases in total), 4 people had previous suicide attempts [56,76,93,98]. In addition, 18 of the 81 cases involved individuals known to have abused alcohol [56,60,65,68,71,78,84,85,89,90,95,112,115–117].

Table S1. Cases of ethylene glycol intoxications reported in the literature.

Reference	Sex	Age [years]	Fatal / non-fatal	Cause of death	Route of intoxication	Suicide/ homicide/ accident	Ethylene glycol concentration [μg/mL]*	Symptoms (selected)	Autopsy results (selected)	Other detected substances	Other information
[49]	M	75	fatal	acute ethylene glycol intoxication and multiple head, trunk and limbs injuries	unknown	homicide	7110 (*ante mortem* blood)<br>152 (*post mortem* blood)<br>100 (heart blood)<br>9570 (cerebrospinal fluid)<br>293 (vitreous humor)	nausea, neurological deterioration, metabolic acidosis	trauma, pathological changes of the heart and kidneys, the presence of calcium oxalate crystals in the kidneys and brain	propylene glycol	the present injuries resulted from blunt force trauma
[56]	M	29	fatal	unknown	consumption of 50% ethylene glycol solution	suicide	unknown	drowsiness, vomiting, metabolic acidosis, seizures	edema of the kidneys, lungs and brain, hyperemia of internal organs, liver steatosis, presence of calcium oxalate crystals in the kidneys and liver	ethanol	treated for depression
	M	39	fatal	unknown	consumption of ethylene glycol solution	suicide	unknown	metabolic acidosis, loss of consciousness, seizures	edema of the kidneys and brain, hyperemia of internal organs, bronchopneumonia and mucopurulent tracheitis and bronchitis, the presence of calcium oxalate crystals in the renal tubules		

	M	64	fatal	unknown	consumption of ethylene glycol solution	suicide	0.3% (*post mortem* urine)		putrefaction, the fingers of the right hand were stained yellow, the same color as the contents of the bottle of ethylene glycol, the presence of calcium oxalate crystals in the renal tubules and liver		suffered from depression, was an alcoholic and had a history of three suicide attempts
	M	41	fatal	ethylene glycol intoxication	unknown	accident	unknown	seizures, hyperventilation, metabolic acidosis	edema of the kidneys and lungs, generalized hyperemia of internal organs, degenerative changes in the liver, presence of calcium oxalate crystals in the renal tubules, fatty degeneration of liver tissue with fibrosis, acute meningitis		abused alcohol
	M	42	fatal	ethylene glycol intoxication	consumption of hand sanitizer liquid	accident	unknown	hypertension, metabolic acidosis, seizures	presence of crystals, calcium oxalate in the kidneys		abused alcohol, addicted to drugs
[93]	M	33	non-fatal	–	consumption of ethylene glycol in the form of an antifreeze	suicide attempt	7060 (blood)		–	ethanol	had a history of two suicide attempts
[57]	M	35	non-fatal	–	consumption of ethylene glycol in the form of an antifreeze	suicide attempt	8500 (serum)	drowsiness, increased breathing rate, presence of calcium oxalate crystals in urine	–		

[58]	M	29	non-fatal	–	unknown	unknown	640 (serum)	hypertension, metabolic acidosis, presence of calcium oxalate crystals in urine	–	cannabinoids	was abusing psychoactive substances
[115]	M	19	non-fatal	–	unknown	unknown	1100 [a]	loss of consciousness, vomiting, apathy, hypertension, presence of calcium oxalate crystals in urine	–		
[112]	M	50	non-fatal	-	unknown	unknown	357 [a]	drowsiness	–	ethanol	abused alcohol
[94]	M	28	non-fatal	–	consumption of ethylene glycol in the form of an antifreeze	suicide attempt	unknown	hypertension, apathy, metabolic acidosis	–		
[95]	M	42	non-fatal	–	consumption of ethylene glycol in the form of an antifreeze	suicide attempt	2840 (serum)	nausea, vomiting	–	ethanol	suffered from depression, hepatitis C, abused alcohol and cocaine
[60]	M	57	fatal	unknown	consumption of ethylene glycol in the form of an antifreeze	suicide	unknown	metabolic acidosis, loss of consciousness, presence of calcium oxalate crystals in kidneys	edema and erosions of the gastrointestinal mucosa, the presence of calcium oxalate crystals in the renal tubules		abused alcohol and had chronic kidney disease
[61]	M	73	non-fatal	–	consumption of ethylene glycol in the form of an antifreeze	suicide attempt	720 (plasma)	presence of calcium oxalate crystals in urine	–	ethanol	present stab wound in the abdominal region (self-inflicted)

[62]	M	41	non-fatal	–	consumption of ethylene glycol solution	suicide attempt	940 [a]	abdominal pain, nausea, vomiting, loss of consciousness, presence of calcium oxalate crystals in urine	–		suffered from depression
[96]	M	16	non-fatal	–	consumption of ethylene glycol in the form of an antifreeze	suicide attempt	1630 [a]	hypertension, tachycardia, loss of consciousness, agonal breath	–		suffered from depression
	F	59	non-fatal	–	consumption of ethylene glycol in the form of an antifreeze	suicide attempt	5110 [a]	drowsiness	–	ethanol	suffered from depression
[97]	F	29	non-fatal	–	consumption of ethylene glycol in the form of an brake fluid	suicide attempt	unknown	impaired consciousness, abdominal pain, vomiting, fever, hypertension	–		
[63]	F	43	non-fatal	–	consumption of ethylene glycol	suicide attempt	147 [a]	ataxia, slurred speech, metabolic acidosis, presence of calcium oxalate crystals in urine	–		suffered from depression

[64]	M	59	fatal	–	consumption of ethylene glycol	suicide or accident	1320 (serum)	dizziness, visual disturbances, loss of consciousness, unresponsiveness to pain stimulus, metabolic acidosis, presence of calcium oxalate crystals in urine	unknown		
[113]	M	47	unknown	–	unknown	unknown	3830 (serum)	hypertension, tachycardia, metabolic acidosis	unknown	benzodiazepines	
[65]	M	56	fatal	unknown	unknown	unknown	425 (serum)	loss of consciousness, presence of calcium oxalate crystals in the urine, metabolic acidosis	bronchiolitis, cerebral encephalomalacia, renal tubular cells degeneration, presence of calcium oxalate crystals in the kidneys tract		abused alcohol
[66]	M	2.5	non-fatal	–	inhalation and consumption of ethylene glycol	accident	370 (serum)	vomiting, apathy, presence of calcium oxalate crystals in urine	–		the child was playing with an unscrewed bottle with antifreeze
[67]	M	19	non-fatal	–	consumption of 200 mL of ethylene glycol	unknown	900 (serum)<br>4000 (urine)	ataxia, drowsiness, slurred speech, presence of calcium oxalate crystals in urine, convulsions	–		
[68]	M	36	fatal	unknown	unknown	unknown	unknown	anuria, oliguria, hypertension	numerous petechiae present on the skin and mucous membrane of the gastrointestinal tract, edema of the kidneys, calcium oxalate crystals present in the kidneys		abused alcohol

[69]	M	27	non-fatal	–	consumption of ethylene glycol in the form of an antifreeze	unknown	145 (blood)<br>80.5 (urine)	disturbance of consciousness, presence of calcium oxalate crystals in urine	–		
[101]	M	33	non-fatal	–	consumption of ethylene glycol	accident	1100 (serum)	loss of consciousness	–		ethylene glycol was found in a whisky bottle
[118]	M	23	non-fatal	–	unknown	unknown	217 (serum)	vomiting, anuria	–		
[105]	M	29	non-fatal	–	consumption of 120 mL of ethylene glycol in the form of an antifreeze	unknown	unknown	agitation	–		
[119]	M	36	non-fatal	–	unknown	unknown	4650 (urine)	loss of consciousness, metabolic acidosis, hypertension, seizures, anuria	–		
[70]	F	36	non-fatal	–	unknown	unknown	2535 (plasma)	nausea, vomiting, hypertension, presence of calcium oxalate crystals in urine, oliguria	–		suffered from depression
	F	38	non-fatal	–	consumption of 240 mL of ethylene glycol in the form of an antifreeze	unknown	3500 (plasma)	drowsiness, hypertension, presence of calcium oxalate crystals in urine, oliguria	–		

[98]	M	23	non-fatal	–	consumption of 400-800 mL of ethylene glycol in the form of a brake fluid	suicide attempt	unknown	confusion	–		had a history of suicide attempts
[114]	F	47	non-fatal	–	unknown	unknown	unknown	drowsiness, hyperventilation, hypothermia, unresponsiveness to pain stimulus	–		suffered from depression
[89]	M	72	fatal	ethylene glycol and methanol intoxication	consumption of a mixture of 80% methanol and 20% ethylene glycol	unknown	unknown	impaired consciousness, vomiting, metabolic acidosis, cold skin, cyanosis	unknown	methanol (in the ingested fluid)	abused alcohol
[106]	M	37	non-fatal	–	consumption of ethylene glycol in the form of an antifreeze	unknown	191 (plasma)	impaired consciousness, metabolic acidosis, brain edema	–		
	F	77	non-fatal	–	consumption of ethylene glycol in the form of an antifreeze	unknown	617 (plasma)	agitation, renal failure, brain edema	–		
[71]	M	54	fatal	unknown	unknown	unknown	7750 (serum)	unresponsiveness, presence of calcium oxalate crystals in urine, metabolic acidosis, anuria	presence of calcium oxalate crystals in tubular lumens, degeneration of the tubules	ethanol	chronic alcoholic
[59]	F	46	non-fatal	–	unknown	unknown	590 (serum)	coma, metabolic acidosis, tachycardia, oliguria	–		abused alcohol
[72]	M	28	non-fatal	–	consumption of ethylene glycol in the form of an antifreeze	suicide attempt	8880 [a]	tachypnoea, presence of calcium oxalate crystals in urine, metabolic acidosis, anuria	–	ethanol, benzodiazepines	suffered from depression

[104]	M	37	non-fatal	–	consumption of food laced with an antifreeze	attempted homicide	unknown	renal failure, facial paralysis, loss of vision and hearing	–		
[107]	M	22	non-fatal	–	consumption of ethylene glycol in the form of an antifreeze	unknown	unknown	vomiting, agitation, tachypnoea, metabolic acidosis	–		suffered from schizophrenia
[73]	M	73	fatal	ethylene glycol intoxication	consumption of 500 ml of “Bluecol” which main ingredient is ethylene glycol	unknown	500 (blood)	disorientation, vomiting, metabolic acidosis	acute gastritis, presence od calcium oxalate crystals in the renal tubules		
[102]	M	30	non-fatal	–	consumption of 100 g of ethylene glycol	accident	3500 (plasma)	consciousness, no dyspnoea	–		
[103]	F	4	non-fatal	–	consumption of an antifreeze containing 41% ethylene glycol	accident	3100 (plasma)	vomiting, drowsiness, metabolic acidosis	–		
[50]	M	60	fatal	ethylene glycol intoxication	unknown	unknown	7800 (blood)<br>10200 (urine)	hypothermia, metabolic acidosis, presence of calcium oxalate crystals in urine, anuria	open tuberculosis, swollen kidneys		
[120]	F	27	non-fatal	–	consumption of a cup of “something blue that goes in cars"	unknown	2551 (serum)	slurred speech, metabolic acidosis, aggressiveness	–		had a suicide attempt 2 months before intoxication
[90]	M	30	non-fatal	–	consumption of 150 mL of an antifreeze containing	unknown	510 (plasma)	coma, metabolic acidosis, anuria	–		suffered from depression

					99% ethylene glycol						
	M	54	fatal	ethylene glycol intoxication	consumption of 200-300 g of ethylene glycol in a form of an aniseed-flavoured alcohol	accident	2500 (plasma)	coma, convulsions, hypothermia, anuria, metabolic acidosis	unknown		chronic alcoholic
[18]	M	36	fatal	ethylene glycol intoxication	unknown	unknown	unknown (0.44 g% of glycolic acid in blood)	coma, tachycardia, oliguria, hypotension, metabolic acidosis, proteinuria	pluriorganic stasis, cerebral, focal cerebral microhemorrhages, pleural effusion, bronchopneumonia, pulmonary edema	ethanol	
[108]	F	28	non-fatal	–	consumption of 400 mL of ethylene glycol	unknown	2480 (serum)	speech difficulties, convulsions, hyperventilation, coma, metabolic acidosis	–		pregnant, after delivery by Caesarean section the neonate exhibited metabolic acidosis and 2206 μg/mL of ethylene glycol in serum
[91]	M	1.5	fatal	ethylene glycol intoxication	consumption of ethylene glycol 3 weeks prior to admission	unknown	unknown	fever, nausea, bloody vomiting, gastrointestinal bleeding, encephalopathy, coma, metabolic acidosis, anuria	increased number of histiocytes with hemophagocytosis		
[111]	M	–	non-fatal	–	consumption of homemade alcohol	unknown	unknown	vomiting, metabolic acidosis	–		

	M	–	non-fatal	–	consumption of homemade alcohol	unknown	unknown	vomiting, metabolic acidosis	–		
	M	–	non-fatal	–	consumption of homemade alcohol	unknown	unknown	vomiting, metabolic acidosis	–		
[74]	M	52	non-fatal	–	aspiration of an antifreeze into mouth several times with spitting it out	accident	unknown	anuria, metabolic acidosis, proteinuria, presence of calcium oxalate crystals in tubular lumens (renal biopsy)	–		
[75]	F	71	fatal	ethylene glycol intoxication	unknown	unknown	1070 (blood)	slurred speech, nausea, vomiting, unresponsiveness, metabolic acidosis, presence of calcium oxalate crystals in urine	unknown		
[76]	M	72	non-fatal	–	consumption of ethylene glycol in the form of an antifreeze	suicide attempt	10700 (serum)	decreased consciousness, metabolic acidosis, presence of calcium oxalate crystals in urine	–	ethanol	suffered from depression with three previous suicide attempts
	M	59	non-fatal	–	consumption of ethylene glycol in the form of an antifreeze	unknown	2545 (serum)	slurred speech, disorientation, metabolic acidosis	–	ethanol	suffered from depression with several previous suicide attempts
[77]		26	non-fatal	–	consumption of an antifreeze containing 95% ethylene glycol	unknown	440 [a]	metabolic acidosis, presence of calcium oxalate crystals in urine, anuria	–		

[108]	M	52	non-fatal	–	consumption of ethylene glycol in the form of an antifreeze	intentional but not suicide	150 (serum)	nausea, vomiting, anuria, metabolic acidosis	–		
[116]	M	60	non-fatal	–	unknown	unknown	322 (blood)<br>1606 (urine)	tachypnoea, metabolic acidosis	–		abused alcohol
[78]	F	45	non-fatal	–	unknown	suicide attempt	79 (serum)	coma, metabolic acidosis, presence of calcium oxalate crystals in urine, anuria, oliguria	–		chronic alcoholic
[79]	M	68	non-fatal	–	consumption of ethylene glycol in the form of an antifreeze	suicide attempt	2523 [a]	slurred speech, confusion, ataxia, metabolic acidosis, presence of calcium oxalate crystals in urine	–		
[80]	F	52	non-fatal	–	consumption of ethylene glycol in the form of an antifreeze	suicide attempt	220 [a]	metabolic acidosis, presence of calcium oxalate crystals in urine	–		
[81]	M	7	non-fatal	–	consumption of ethylene glycol in the form of an antifreeze	unknown	3900 (serum)<br>13600 (urine)	blurred speech, metabolic acidosis, presence of calcium oxalate crystals in urine	–		
[82]	M	78	non-fatal	–	unknown	unknown	540 (serum)	tachycardia, lethargy, disorientation, metabolic acidosis, oliguria, presence of calcium oxalate crystals in urine	–		
[99]	F	12	non-fatal	–	consumption of 250 g of ethylene glycol	suicide attempt	880 (serum)	unremarkable, no evidence of intoxication	–		

[83]	M	30	non-fatal	–	consumption of ethylene glycol	unknown	40 (serum)	headache, vomiting, diplopia, anuria, metabolic acidosis, presence of calcium oxalate crystals in tubular lumens (renal biopsy), coma	–		
[84]	M	61	non-fatal	–	unknown	suicide attempt	1270 (blood)	unresponsiveness, metabolic acidosis, oliguria, presence of calcium oxalate crystals in urine	–		suffered from depression, abused alcohol
[117]	M	53	non-fatal	–	unknown	unknown	144 (serum)	dizziness, slurred speech, nausea, vomiting, disorientation, tachycardia, metabolic acidosis, proteinuria, presence of oxalate crystals in tubules (renal biopsy)	–		abused alcohol
	M	49	non-fatal	–	unknown	unknown	unknown	rash, slurred speech, sleepiness, dizziness, anuria, presence of oxalate crystals in tubular lumina (renal biopsy)	–		had a suicide attempt 5 years before
[78]	M	46	non-fatal	–	consumption of ethylene glycol in the form of an antifreeze	unknown	unknown (1230.7 µg/mL of glycolic acid in urine)	metabolic acidosis, renal failure	–	cannabis, benzodiazepines	
[92]	M	28	fatal	unknown	consumption of ethylene glycol in the form of 500 mL of an antifreeze	unknown	6792 (serum)	aggressiveness, dilated pupils, metabolic acidosis	unknown		consumption of 60 tablets of co-proxamol and a bottle of whisky together with an antifreeze

	M	39	fatal	unknown	consumption of ethylene glycol in the form of 500-2000 mL of an antifreeze	unknown	3700 (serum)	unconsciousness, unresponsiveness, dilated pupils, metabolic acidosis	unknown		
	F	27	non-fatal	–	consumption of ethylene glycol in the form of 500 mL of an antifreeze	suicide attempt	3000 (serum)	unconsciousness	–		consumption of 2-3 pints of beer together with an antifreeze
[97]	M	51	non-fatal	–	consumption of ethylene glycol in the form a brake oil	unknown	unknown	shortness of breath, oliguria, metabolic acidosis	–		
[85]	M	62	fatal	multiorgan failure	consumption of ethylene glycol	unknown	2000 (blood)	cardiac arrhythmia, respiratory insufficiency	presence of calcium oxalate crystals in kidneys and brain	ethanol	
	F	83	fatal	unknown	unknown	unknown	approx. 2000 (blood)	unknown	arteriosclerosis with ulceration and calcifications, heart enlargement, presence of calcium oxalate crystals in kidneys and brain		was an alcoholic
	M	47	fatal	unknown	consumption of ethylene glycol in the form of an antifreeze	unknown	approx. 2000 (blood)	unknown	presence of calcium oxalate crystals in kidneys and brain		was an alcoholic
[121]	F	46	non-fatal	–	ingestion of ethylene glycol	suicide	unknown	slurred speech, left arm paresthesia, vision changes, metabolic acidosis, tachypnea	–		
[86]	F	35	non-fatal	–	ingestion of 500 mL of an antifreeze	unknown	unknown	isochoric pupils, unremarkable brainstem reflexes, metabolic acidosis, presence of	–	benzodiazepines	was pregnant, had a previous suicide attempt

								calcium oxalate and glycolate crystals in urine			
[87]	M	57	non-fatal	–	possible antifreeze ingestion	unknown	1120 (blood)	slurred speech, hypothermia, bradycardia, metabolic acidosis	–		
[88]	F	19	non-fatal	–	ingestion of a radar cooling fluid	accident	450 (blood)	hypothermia, tachycardia, metabolic acidosis, agitation, presence of calcium oxalate crystals in urine	–		were soldiers, 9 more people got intoxicated (no details given)
	M	27	non-fatal	–	ingestion of a radar cooling fluid	accident	200 (blood)	hypothermia, metabolic acidosis, presence of calcium oxalate crystals in urine	–		
[122]	F	28	non-fatal	–	unknown	suicide attempt	8400 (blood)<br>9400 (urine)	hypothermia, tachynoea, tachycardiametabolic acidosis, anuria			a farewell letter was found, had previous suicide attempts

M – male; F – female; * - unless otherwise stated; [a] – the biological material was not specified

S2. Mass spectrometry spectra aquired in scan mode and product ion scan mode

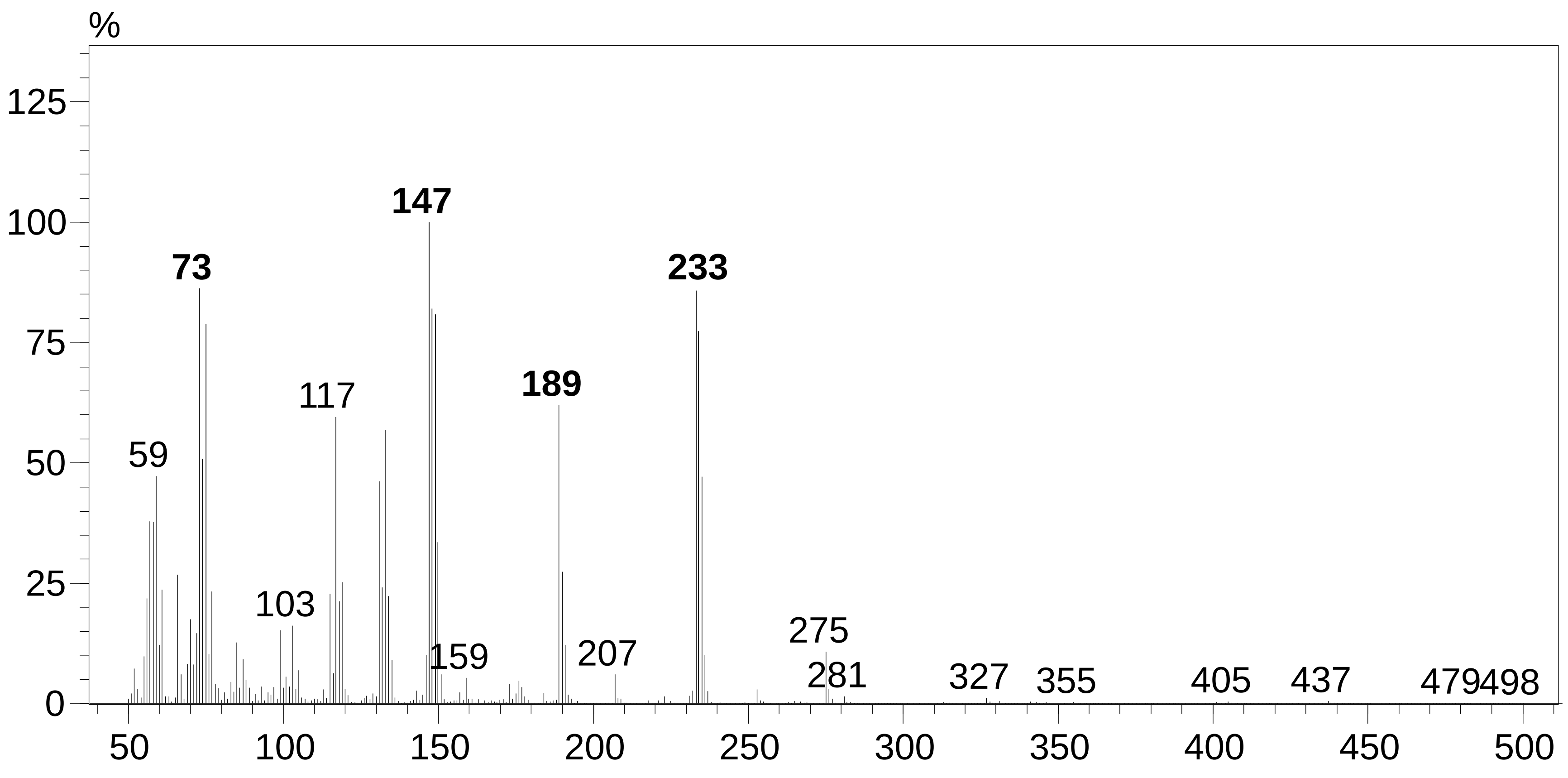

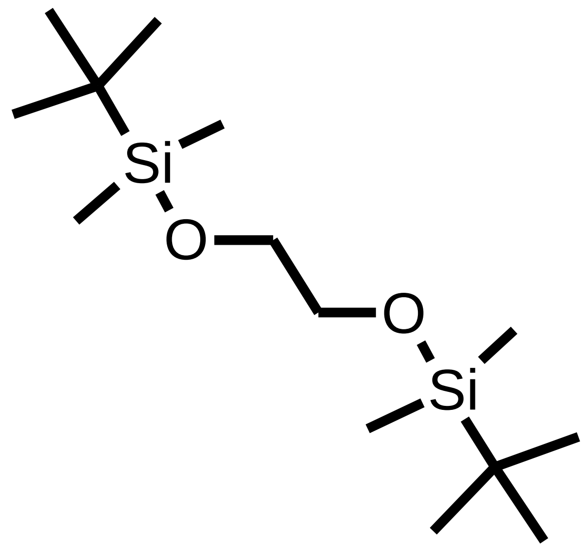

Ethylene glycol-bis-TBDMS

$M^+ = 290$ m/z

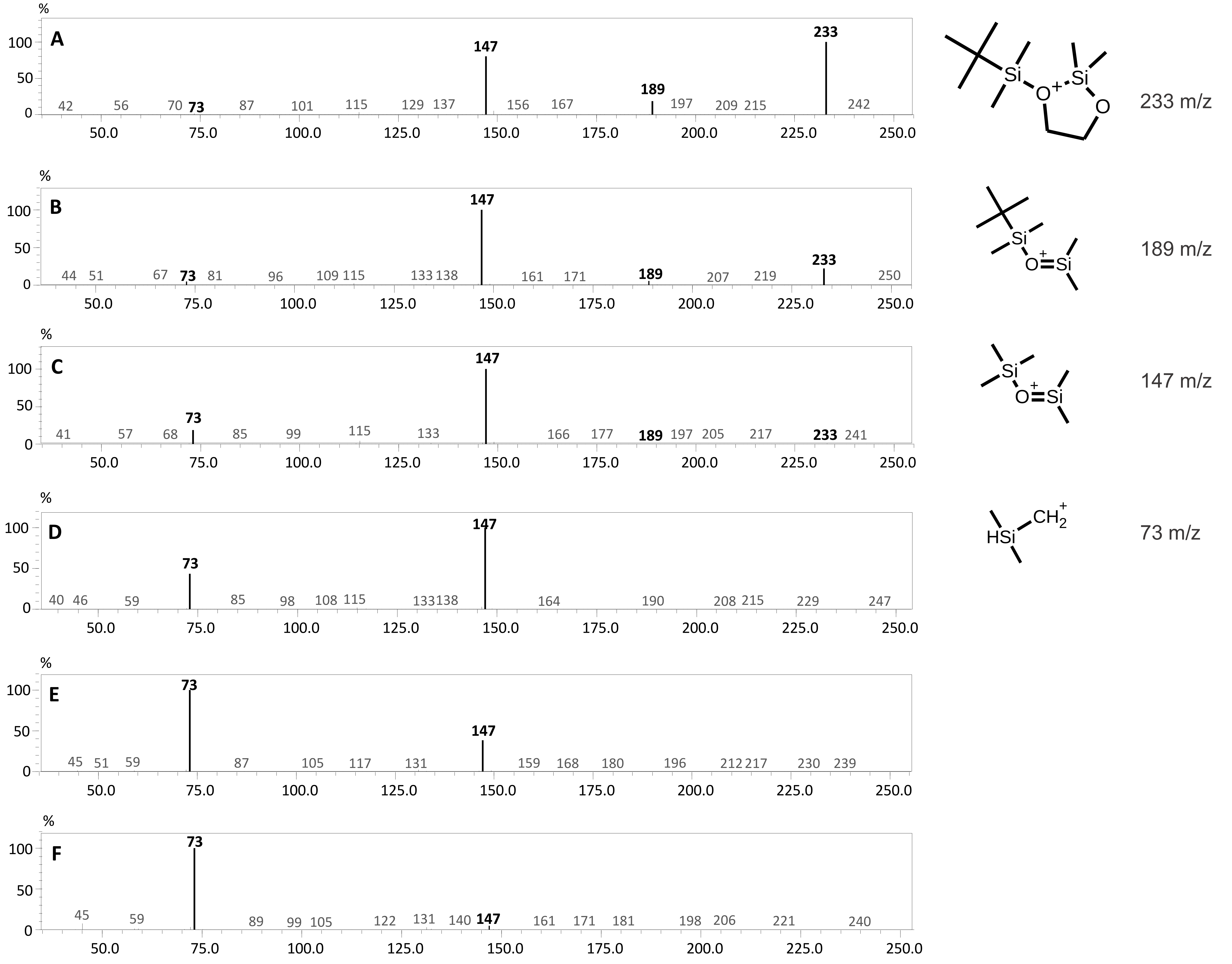

A: 2 V; **B:** 5 V; **C:** 10 V; **D:** 15 V; **E:** 25 V; **F:** 35 V

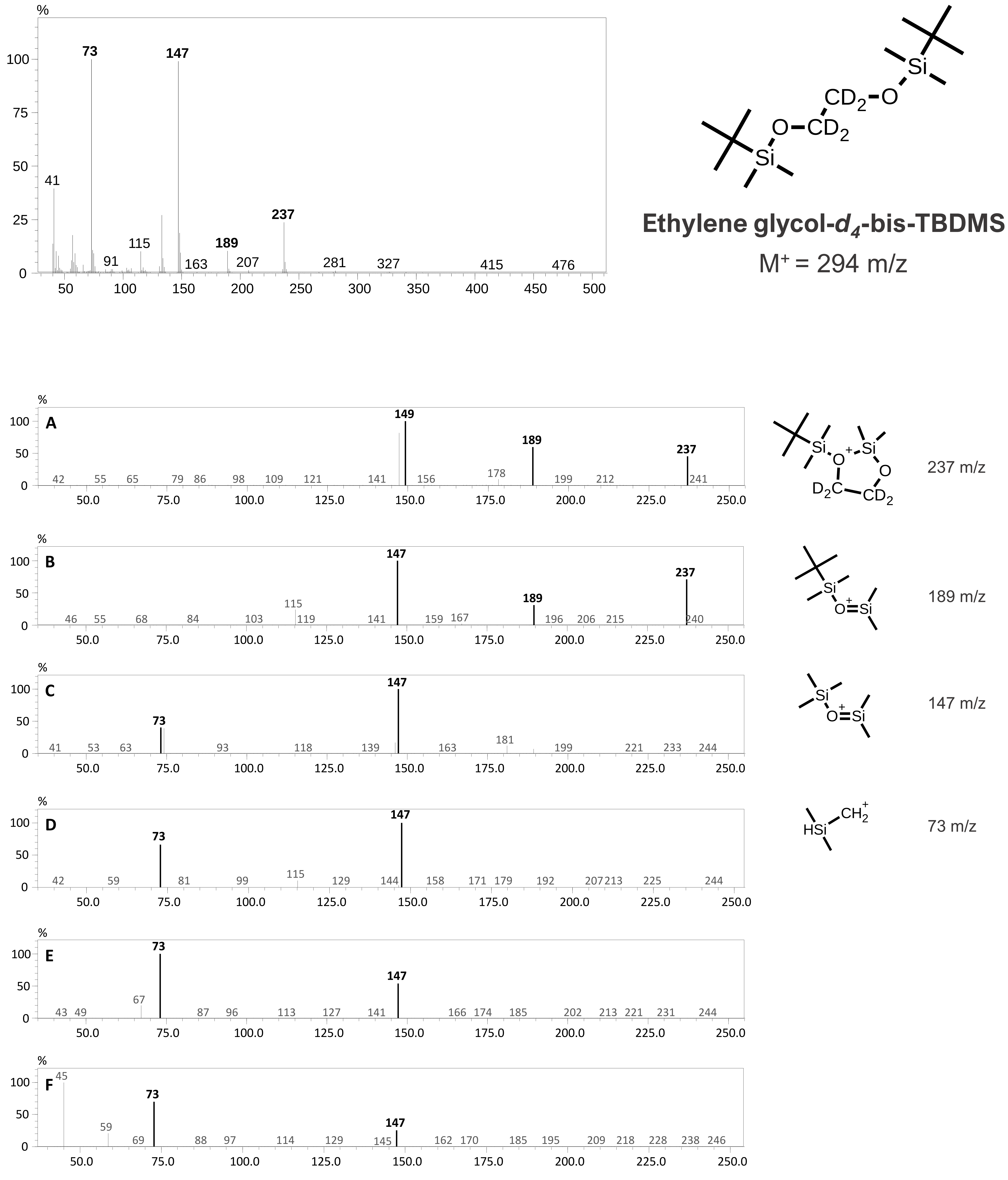

A: 2 V; **B:** 5 V; **C:** 10 V; **D:** 15 V; **E:** 25 V; **F:** 35 V

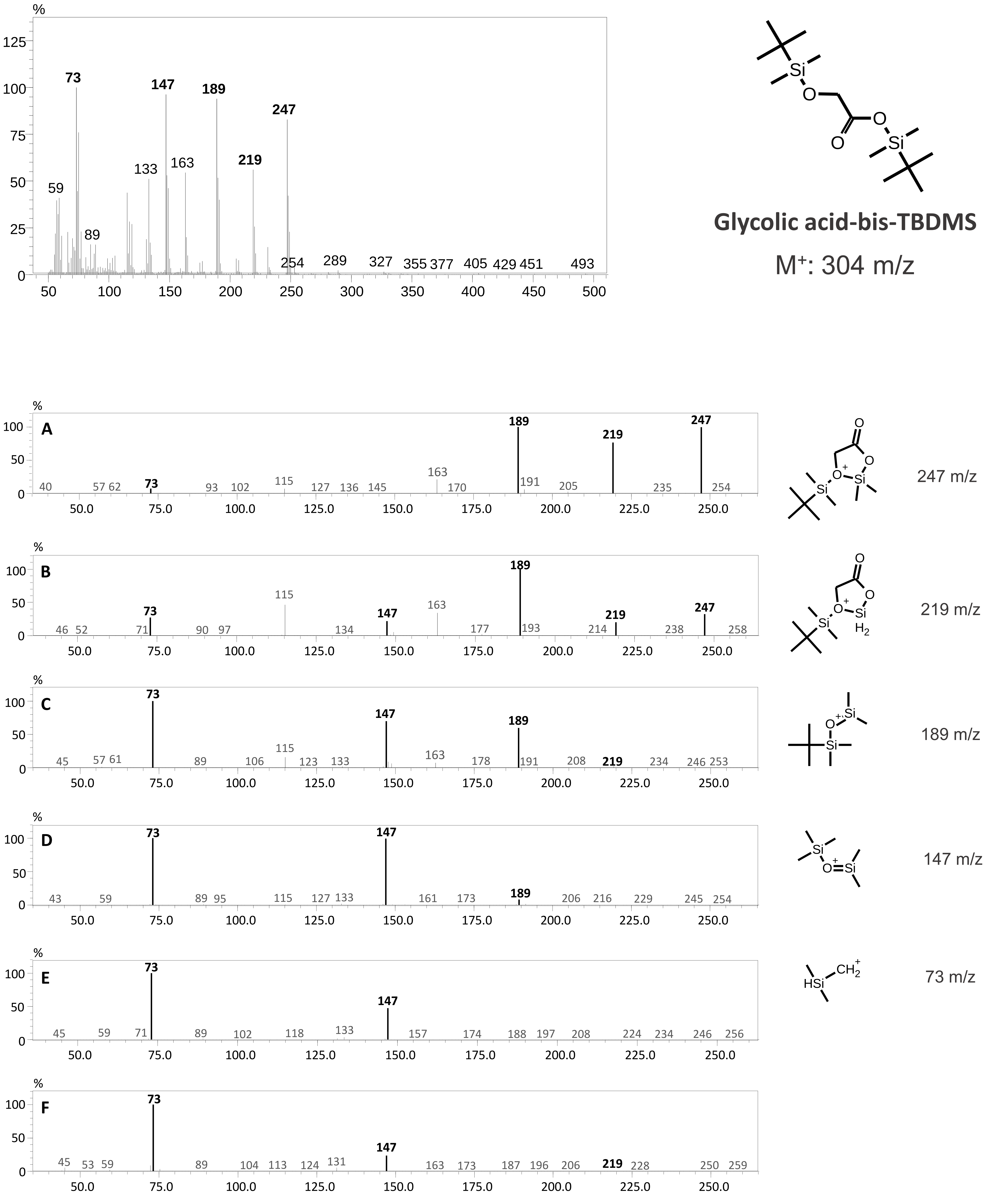

A: 2 V; **B:** 5 V; **C:** 10 V; **D:** 15 V; **E:** 25 V; **F:** 35 V

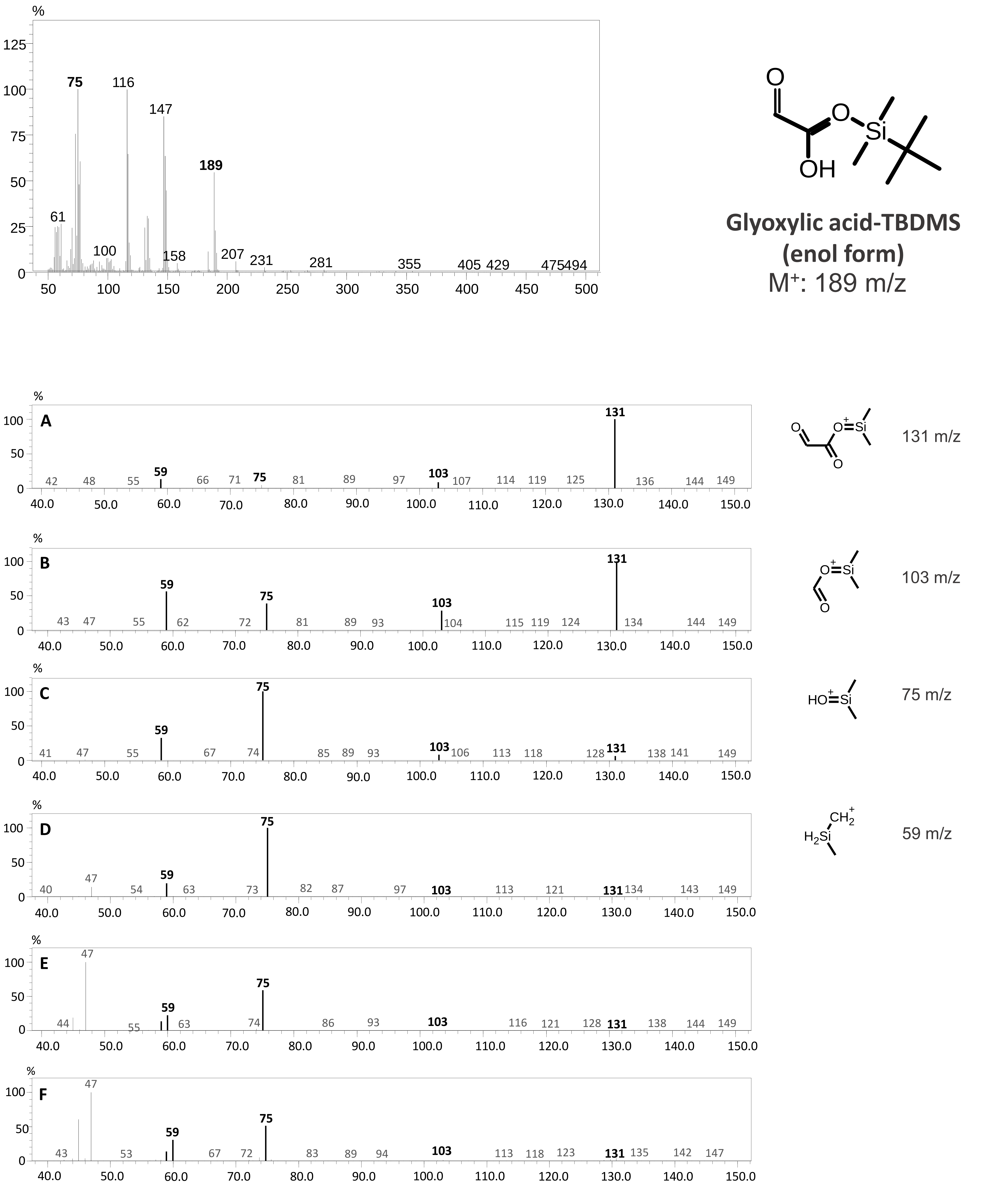

A: 2 V; **B:** 5 V; **C:** 10 V; **D:** 15 V; **E:** 25 V; **F:** 35 V

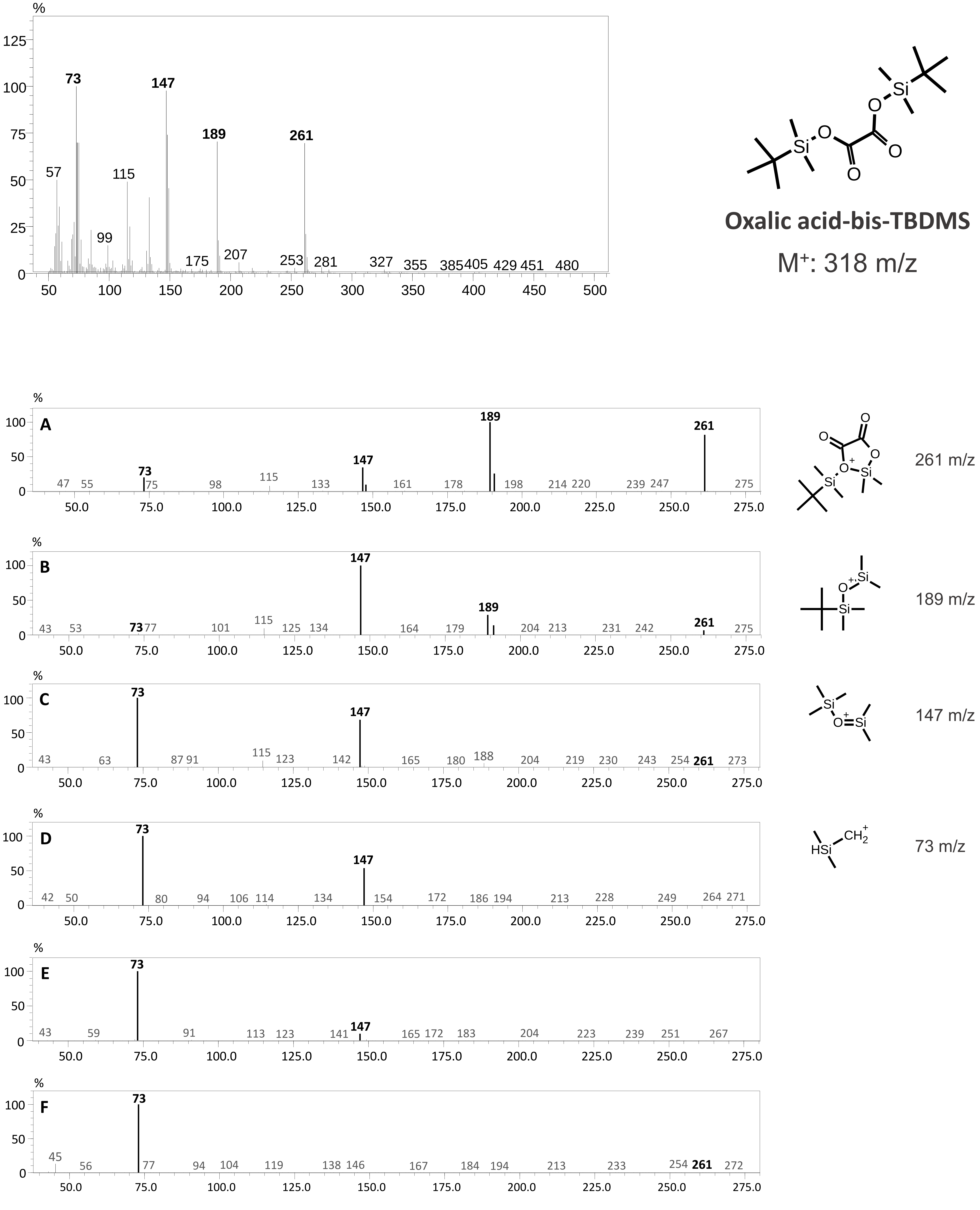

A: 2 V; **B:** 5 V; **C:** 10 V; **D:** 15 V; **E:** 25 V; **F:** 35 V

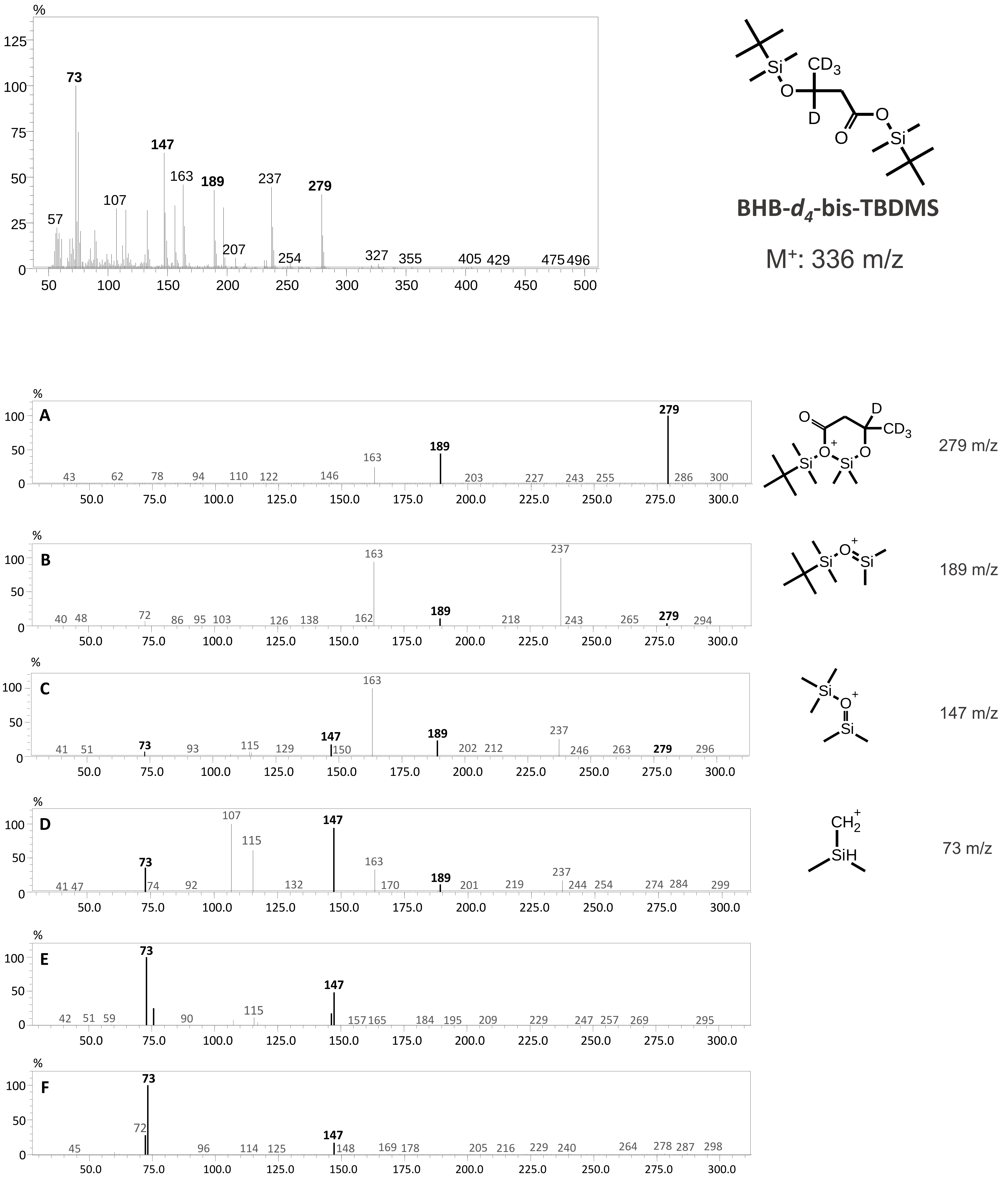

A: 2 V; **B:** 5 V; **C:** 10 V; **D:** 15 V; **E:** 25 V; **F:** 35 V

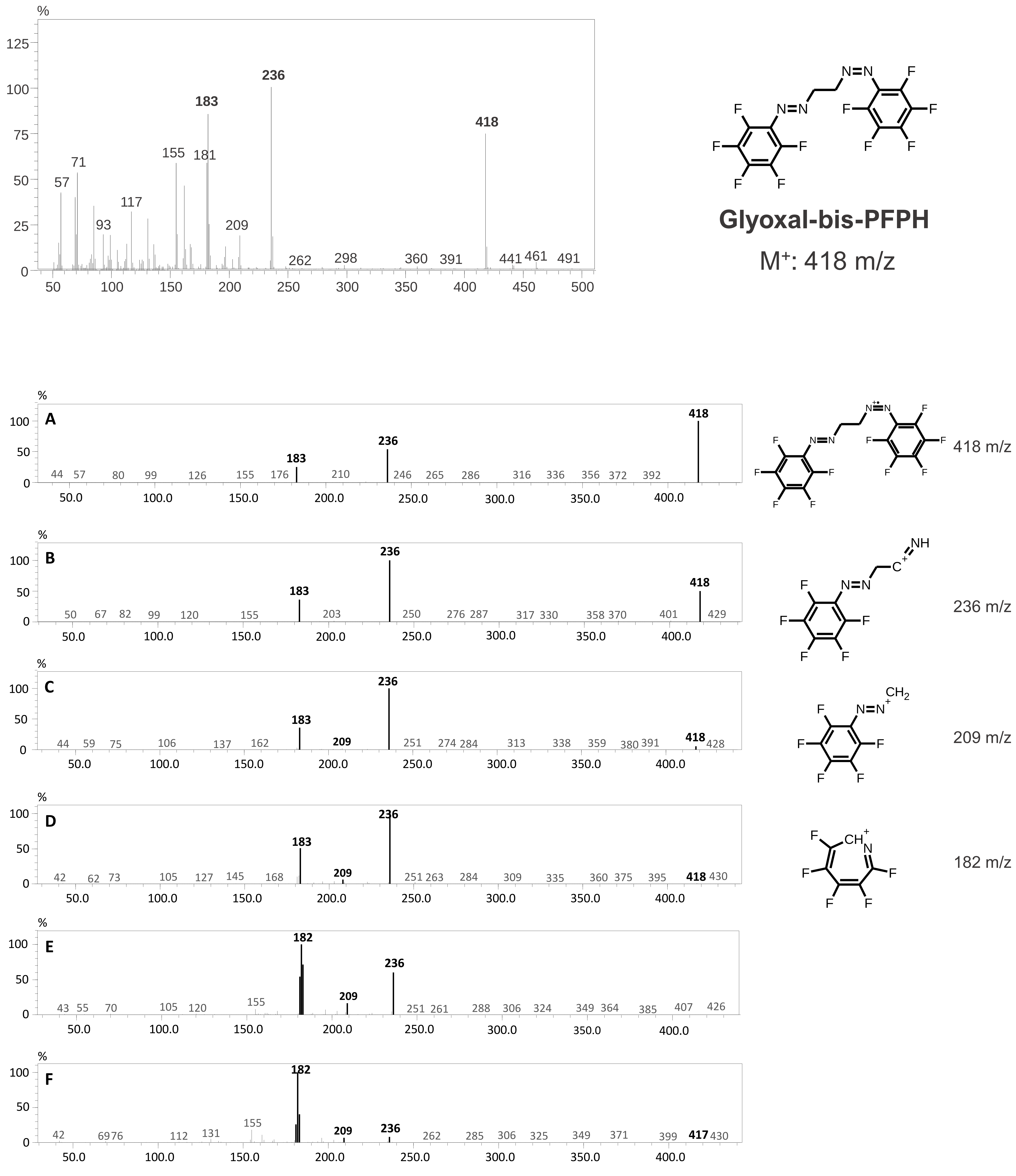

A: 2 V; **B:** 5 V; **C:** 10 V; **D:** 15 V; **E:** 25 V; **F:** 35 V

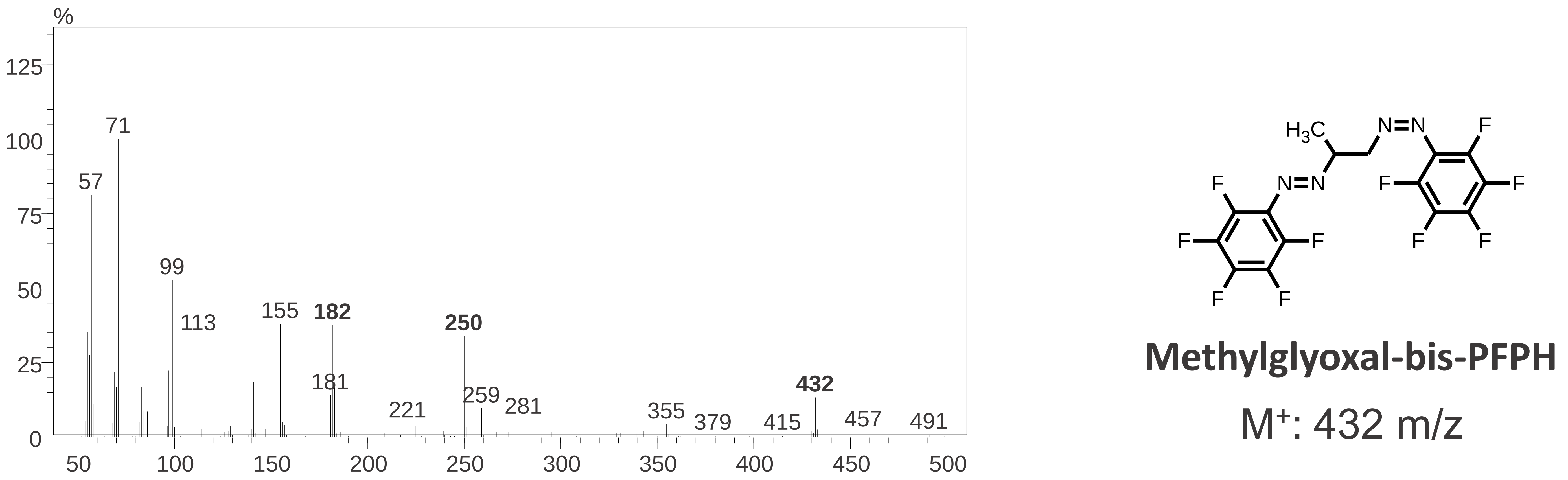

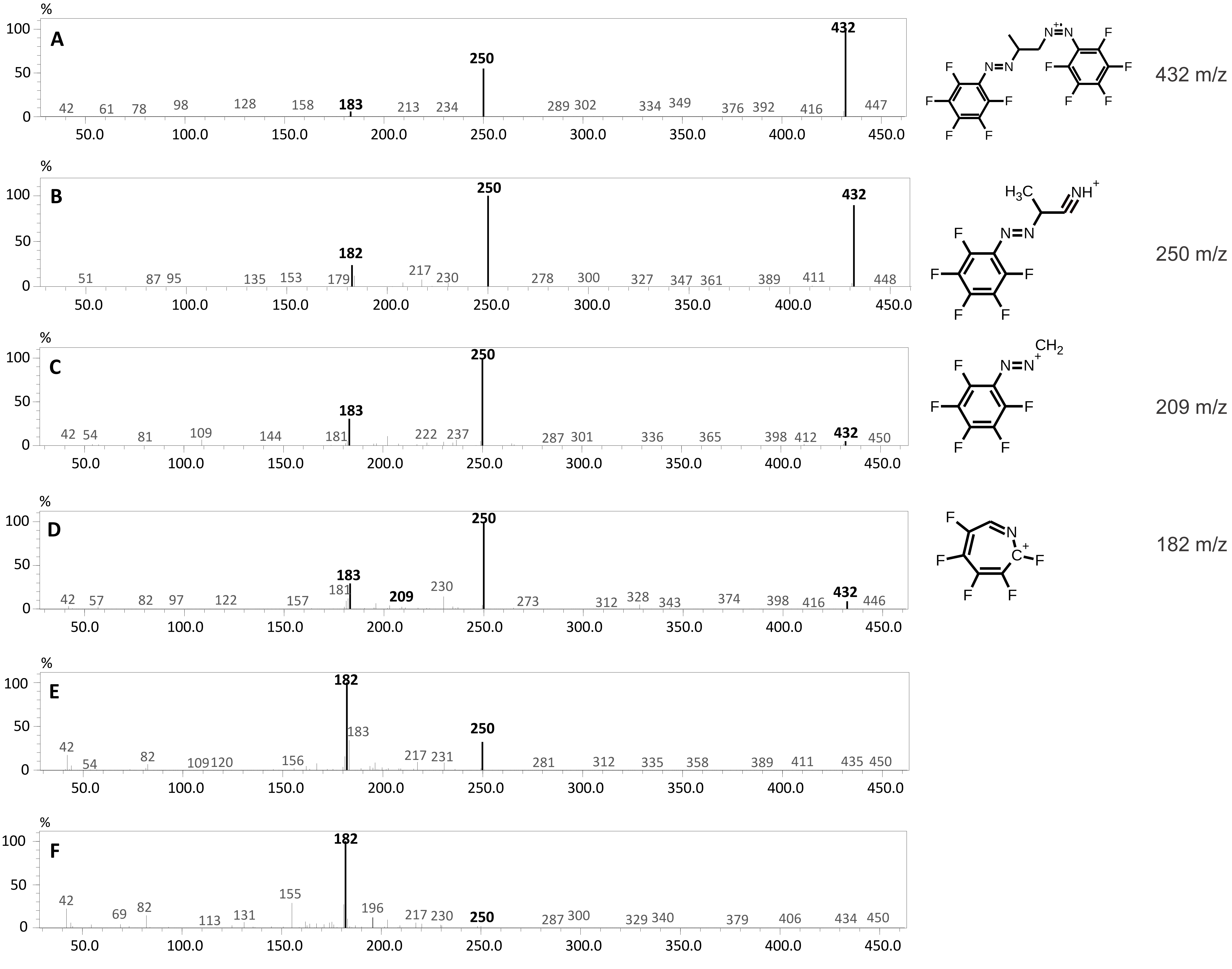

A: 2 V; **B:** 5 V; **C:** 10 V; **D:** 15 V; **E:** 25 V; **F:** 35 V

Table S3. Parameters of the method for quantification of ethylene glycol and its metabolites in urine samples.

Substance	Calibration curve: The coefficient of determination (R^2)	Validation parameters: Concentration level [µg/mL]	Intraday: Precision RSD [%]*	Intraday: Accuracy RE [%]*	Interday: Precision RSD [%]*	Interday: Accuracy RE [%]*	Recovery [%]*	Matrix effect [%]*	Dilution effect RE [%]*
Ethylene Glycol	0.9998	20	5.1	-2.0	2.5	5.8	97.1	-2.9	–
		100	3.2	-2.1	3.9	-3.2	99.4	-0.6	–
	0.9999	450	4.7	0.5	5.2	-2.4	98.5	-1.5	–
		2500	1.6	1.1	1.7	2.5	100.9	0.9	8.5
Glyoxal	0.9996	0.5	8.3	-3.2	7.2	1.0	99.5	-0.5	–
		5	7.9	0.9	6.1	-2.6	102.8	2.8	–
		50	10.2	-0.5	5.5	0.7	98.9	-1.1	7.1
Glycolic acid	0.9998	500	6.3	-3.4	8.3	7.1	99.9	-0.1	–
		1500	5.5	0.9	10.2	3.4	103.3	3.3	–
		5000	11.6	-1.8	7.9	5.9	101.2	1.2	5.5

BHB – 3-hydroxybutanoic acid; LLOQ – lowest limit of quantification; RSD – relative standard deviation; RE – relative error; S/N – signal to noise ratio * (n = 5)

TIC chromatogram

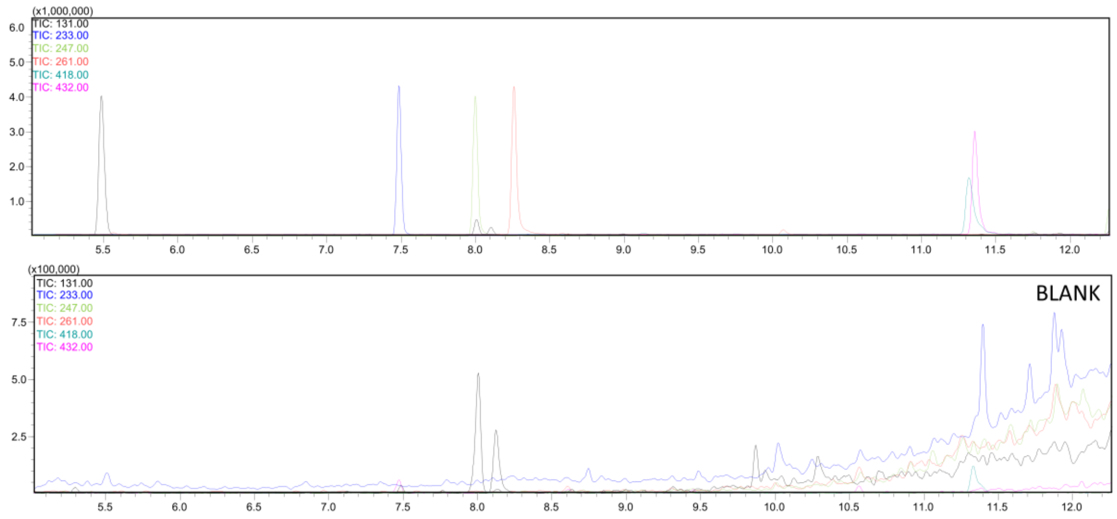

Supplement: Supplementary file 1 [file jox-14-00065-s001.zip › jox-3069811-supplementary.pdf]
